# Network formation and dynamics among multi-LLMs

**DOI:** 10.1093/pnasnexus/pgaf317

**Published:** 2025-12-02

**Authors:** Marios Papachristou, Yuan Yuan

**Affiliations:** Department of Information Systems, W.P. Carey School of Business, Arizona State University, 400 E Lemon St, Tempe, AZ 85281, USA; Department of Computer Science, Cornell University, 107 Hoy Rd, Ithaca, NY 14853, USA; Graduate School of Management, UC Davis, 540 Alumni Ln, Davis, CA 95616, USA

**Keywords:** network formation, large language models, simulations, networks, agent-based modeling

## Abstract

Social networks shape how humans form opinions, exchange information, and organize collectively. As large language models (LLMs) become embedded in social and professional environments, it is critical to understand whether their interactions resemble human network dynamics. We introduce a framework to study the network formation behaviors of multiple LLM agents and benchmark them against human decisions. Across synthetic and real-world settings, including friendship, telecommunication, and employment networks, LLMs reproduce core microlevel principles (preferential attachment, triadic closure, and homophily), and macrolevel properties (community structure, small-world effects). Their emphasis on these principles adapts to context: for example, LLMs favor homophily in friendship networks but heterophily in organizational settings, mirroring patterns of social mobility. A controlled survey shows strong alignment between LLM and human link-formation decisions. These results highlight LLMs’ potential as tools for social simulation and synthetic data generation, while underscoring risks of bias and fairness in AI systems that interact with human networks.

Significance Statement Our study examines the network formation behaviors of multiple large language models (LLM) agents, scrutinizing key principles including preferential attachment, homophily, triadic closure, community structure, and the small-world phenomenon—fundamental elements that shape human social networks. We find that LLMs not only mimic these principles but do so with a degree of sophistication that closely aligns with human behaviors, illuminating their potential to replicate complex network dynamics. This insight broadens our understanding of LLMs’ capabilities, paving the way for applications in network science and social sciences, as well as practical applications such as chatbot development, personal assistant technologies, and synthetic dataset generation, where emulating human-like network behaviors is paramount.

## Introduction

Recent progress in large language models (LLMs), such as GPT (1–3), has enabled their integration into real-life applications. Understanding how these models act in social settings is essential to ensure alignment with human expectations, prevent risks like biased or noncooperative behavior (4), and maximize societal benefits. Researchers have begun applying social science methodologies—including laboratory-style experiments (5–8), agent-based modeling (9–14), and qualitative methods (15)—to study LLMs, shedding light on their interpretability and potential for social science applications (6, 16–18).

Because social networks structure human behavior, preferences, and information diffusion (19–23), it is crucial to understand whether LLMs mirror human principles of network formation (18, 24, 25). Recent studies show that collectives of LLMs can exhibit linguistic conventions (26, 27) and even the friendship paradox (28), underscoring their promise for social simulation (13, 25, 27, 29).

Our work complements these efforts by focusing directly on network formation. We ask: *Which complex social phenomena emerge from interactions between multiple LLMs?* Using both synthetic and real-world networks, we analyze microlevel principles (preferential attachment (30), triadic closure (31), and homophily (32)) and macrolevel properties (community structure (33) and small-world effects (34, 35)).

Our results demonstrate that in synthetic settings, LLMs consistently exhibit human-like behaviors, including preferential attachment, homophily, and triadic closure, resulting in emergent community structures and small-world patterns. In real-world contexts, their preferences adapt: in friendship networks, they emphasize homophily and triadic closure, in telecommunications networks, homophily and preferential attachment dominate, and in company networks, employees preferentially connect to managers, reflecting human social mobility. Thus, LLMs not only capture fundamental principles but also adjust strategies across environments, echoing human context-specific behavior.

These findings carry important implications. First, LLMs offer a novel approach to agent-based modeling, in terms of providing realistic and flexible simulations without the need for hard-coded heuristics. This enables managers, policymakers, and researchers to test interventions in silico before deployment in costly or sensitive real-world settings. Second, LLMs can generate synthetic datasets that replicate key social network properties while preserving privacy. This is especially valuable in domains such as organizational or healthcare networks, where data access is restricted.

Overall, our study positions LLMs as powerful tools for understanding, simulating, and shaping social systems, offering opportunities for both advancing theory and practical applications in business, governance, and technology design.

## Results

In this study, we investigated whether LLMs exhibit fundamental principles of network formation observed in human social networks. By simulating multiple LLM agents acting independently within separate conversational threads, we examined their behaviors in decision-making scenarios involving network connections. We focused on three microlevel network principles—preferential attachment, triadic closure, and homophily—and two macrolevel phenomena—community structure and the small-world effect. To assess the robustness of our findings, we varied the temperature settings of different LLM models, temperatures, and prompting (see Materials and methods). Finally, we extended our analysis to real-world networks, including a social media friendship network, a telecommunication network, and a company collaboration network, to compare the network formation preferences between LLMs and humans.

### Microlevel properties

#### Principle 1: preferential attachment

Preferential attachment is a fundamental concept in network science, illustrating how nodes in a network acquire connections over time, resulting in a scale-free degree distribution characterized by a few highly connected nodes (30, 36).

To test if LLM agents exhibit preferential attachment, we simulated network growth by sequentially adding nodes to an initially empty network. Each new node was prompted with information about existing nodes, and the person to connect with was decided. We generated networks with n=200 nodes to observe meaningful degree distributions. Note that we provide the full network structure in the prompt, so models are not inherently biased toward forming links with the highest-degree nodes.

**Fig. 1.**
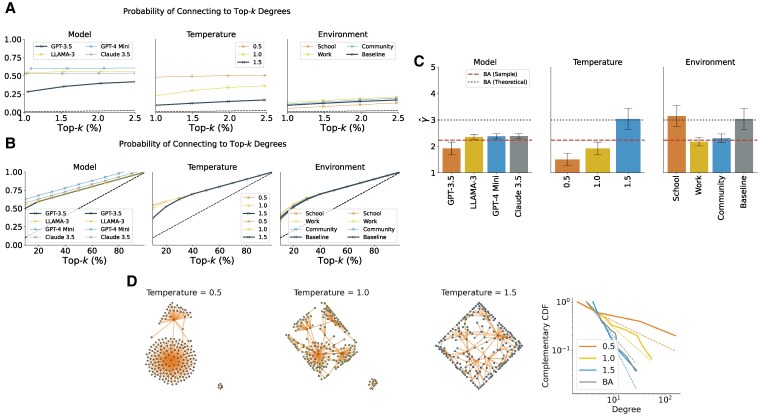
Results for principle 1 (preferential attachment) The multi-LLM setup was given neighborhood information $\left\{N_{\;j,t}\;:\;j\in V_{t}\right\}$. A, B) Probability of connecting to top-*k*-degree nodes for varying model (temperature is fixed to 1.0 and environment to baseline), temperature (model fixed to GPT-3.5 and environment to baseline), and environment (model fixed to GPT-3.5 and environment temperature to 1.5) for networks generated according to principle 1 with n=200 nodes. A) The whole range of *k* is shown, and B) the top 1–2.5% nodes is shown. C) Power law exponents and standard errors for varying model, temperature, and environment. D) Simulated networks. Power-law degree distributions are evident (P>0.5, K–S test), with the networks at a temperature of 1.5 closely resembling the Barabási–Albert model (P>0.1, K–S test) for GPT-3.5 agents.

On a microscale, Fig. 1 illustrates the probability of connecting to a top-*k* node as a function of its degree percentile (k/n). To demonstrate the tendency toward preferential attachment, we compare these probabilities to a null model assuming random connections (represented by dashed lines), where the likelihood of connecting to a top-*k* node is k/n. Our findings reveal that all models prefer connecting to nodes of higher degree. Notably, GPT-3.5 exhibits a weaker preference, while other, arguably more capable models, show an even stronger inclination toward preferential attachment. Using GPT-3.5 as an example, we examine the effect of temperature—a parameter that controls the variability of model output—on this tendency. At lower temperatures, the model makes fewer stochastic choices and, as a result, is more likely to connect to high-degree nodes. We also vary the prompt to explore the influence of environment, “contextual settings such as school, work, or community. The results show slight variations compared to the baseline (GPT-3.5 with temperature = 1.5), yet the tendency for preferential attachment persists across environments. In all cases, the observed curves lie above the null model, underscoring the presence of preferential attachment.

Next, we investigate the degree distribution of the resulting graphs. As shown in Fig. 1, the resulting networks display a pattern where a few nodes have many connections while most have few, indicative of a scale-free distribution, with form $\pi (d)\propto d^{-\gamma }$, where γ>1.

We estimated the exponent *γ* for different models and temperatures. Our analysis reveals several notable patterns in the networks generated by LLM agents under different conditions. First, models newer than GPT-3.5 exhibit a slightly larger $\hat{\gamma }$ than GPT-3.5. This implies that these models display a stronger tendency toward preferential attachment and the formation of hubs. Second, as the temperature increases, the power-law exponent $\hat{\gamma }$ generally becomes larger. This indicates that higher temperatures introduce more variance in node connectivity, leading to degree distributions with heavier tails. Third, the environmental context significantly affects the value of $\hat{\gamma }$. For example, when the network is framed within a “school” environment, the exponent increases, suggesting a more uniform distribution of connections and fewer highly central nodes.

Finally, while the prompts we have utilized thus far have provided the model with the complete existing network structure, we also explore an alternative scenario: what happens if agents are supplied solely with the degree of other alternatives, without access to the network’s full structure? As detailed in the SI Appendix, we find that limiting agents to degree information alone also leads to notable structural differences in the networks that emerge (cf. SI Appendix). Thus, degree information alone yields more restrictive structures than providing the agents with the full topological information (i.e. the neighbors).

The findings highlight the practical potential of LLMs in modeling complex networks, such as social, economic, or biological systems, by leveraging their ability to simulate preferential attachment and scale-free distributions. These models can be used to study real-world phenomena like information diffusion, hub formation, or connectivity patterns under varying conditions. Additionally, the sensitivity of network structures to parameters like temperature and context underscores the importance of prompt design in steering outcomes, making LLMs versatile tools for tailored simulations.

#### Principle 2: triadic closure

The second microlevel principle we examine is triadic closure, which posits that individuals are more likely to form connections with friends of friends, thus creating closed triads in the network. This process strengthens network structure and cohesion, grounded in the idea that two nodes are more likely to connect if they share a common neighbor (31, 37).

To investigate triadic closure, we employ an assortative stochastic block model (SBM) (38) to create an initial network $G_{1}$ with *n* nodes divided into two equal-sized clusters *A* and *B*. Connections within each cluster are formed with a probability of 0.5, while intercluster connections occur with a probability of 0.1. This setup mirrors our assumption that nodes within the same cluster are more inclined to connect due to a higher number of shared neighbors. In subsequent time steps, we then examine each node *i*, considering the intersection of neighborhoods of *i*’s nonneighbors.

We conducted 10 simulations with n=50 nodes to facilitate clear visualization and ensure statistical significance.

**Fig. 2.**
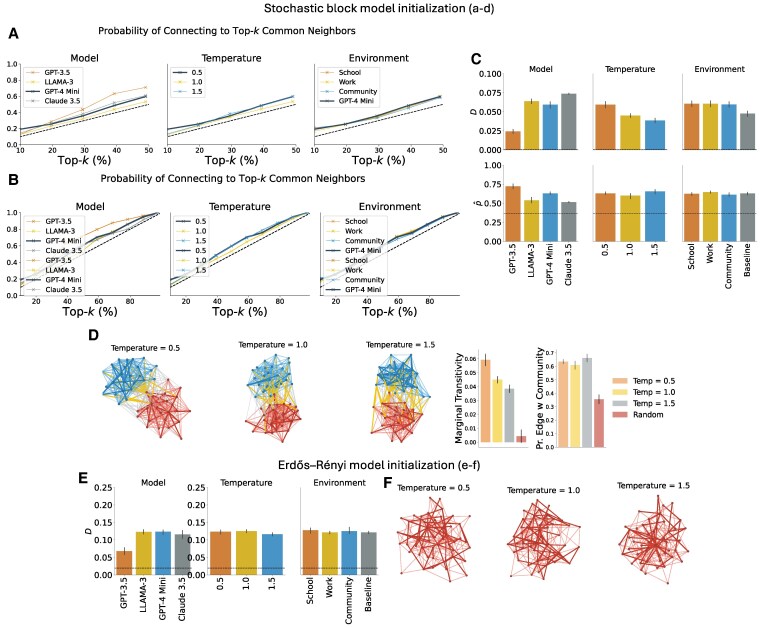
Results for principle 2 (triadic closure). A, B) Probability of connecting to top-*k* nodes (in terms of common neighbors) for varying model (temperature is fixed to 1.0 and environment to baseline), temperature (model fixed to GPT-4 Mini and environment to baseline), and environment (model fixed to GPT-4 Mini and environment temperature to 0.5) for networks generated according to principle 2 (n=50, 10 simulations for each model, environment and temperature). The dotted diagonal line corresponds to the null model, where connections are made at random. A) Top-*k* for *k* for *k* in the range 10–50% is shown, and B) top-*k* for *k* in the range of 10–100% is shown. C) Marginal transitivity (*D*) and probability of an edge within a community ($\hat{p}$) for networks generated according to principle 2 in different models, temperatures, and environments. The dotted line corresponds to the random null model. D) The figure shows the resulting networks created by GPT-4 Mini, according to principle 2, when the intersection of the neighborhoods of the query node and each alternative is provided, and comparison of the metrics *D* and $\hat{p}$ with the random null model. The nodes belong to two groups and the newly created intercluster edges are displayed. E, F) Marginal transitivity (*D*) and network instances when the initial network is an Erdös–Rényi graph with n=50 and p=0.1.

On a microscale, Fig. 2 illustrates the probability of connecting to a top-*k*-percentile node as a function of the number of common neighbors. The dashed lines represent the results of null models, where connections are chosen randomly, which corresponds to the probability of connecting to a top-*k* percentile node in terms of the common neighbors being k/n. Our findings reveal that, across all models, there is a consistently higher probability of forming links with nodes that share more common neighbors. Unlike the behavior observed in preferential attachment, temperature does not appear to impact this probability severely. This tendency to form links with nodes that have more common neighbors is consistent across various contexts, including school, work, and community environments. These results suggest that the triadic closure tendency is a robust phenomenon, persisting across different model families, configurations, and environments.

Then, for evaluating triadic closure on the network (macroscopic) level, we utilize two metrics: *marginal transitivity* and *probability of edge formation within the same community*. Marginal transitivity (*D*) represents the change in the ratio of closed triangles to all triads, transitioning from the SBM-generated network $G_{1}$ to the final network $G_{T}$ after T=50 iterations:


$$D=3\times \frac{\text{\# triangles}(G_{T})}{\text{\# triads}(G_{T})}-3\times \frac{\text{\# triangles}(G_{1})}{\text{\# triads}(G_{1})}.$$

A large positive *D* indicates a strong triadic closure tendency.

As we investigate under SBM, the same community membership indicates more open triads being closed.

Marginal transitivity (*D*), presented in Fig. 2, demonstrates a statistically significant increase across all models, temperatures, and environments, underscoring the robust nature of triadic closure.

In Fig. 2, sample networks from GPT-3.5 are displayed, with the upper panel showing networks where the entire structure is provided and the lower panel showing those with only common neighbor numbers provided. Nodes are color-coded to indicate their cluster memberships in the SBM, with red and blue edges within clusters and orange edges between clusters. Newly formed edges are highlighted with thicker lines.

Finally, to eliminate the possibility that the results are due to structural bias from the initial structure (SBM), we note that we can obtain the same results when we start from a more “neutral” initial topology. Specifically, we get the same results and an even stronger effect for the marginal transitivity (*D*) by starting from a sparse Erdös–Rényi graph with n=50 nodes and P=0.1. We find that, across or models, temperatures and variations of the prompts, the resulting network exhibits higher marginal transitivity (*D*) compared to the random null model, which makes connections at random starting from the same Erdös–Rényi graph, and the results are statistically significant (cf. Fig. 2; P<0.001; *t*-test comparing the marginal transitivity of the resulting LLM-generated networks and the random null model).

In summary, these findings show that most LLMs exhibit a consistent tendency for triadic closure across various configurations, temperatures, and environments. This behavior mirrors human network dynamics, highlighting the models’ ability to simulate realistic social and structural networks and reinforcing their alignment with social principles observed in real-world communities.

#### Principle 3: Homophily

Homophily is the tendency for nodes with similar attributes to connect (32). To test whether LLMs exhibit this behavior, we generated networks of n=50 nodes with randomly assigned features (location, hobby, and favorite color), and asked each node to form up to δ=5 links based on others’ attributes. We evaluated outcomes using assortativity coefficients.

**Fig. 3.**
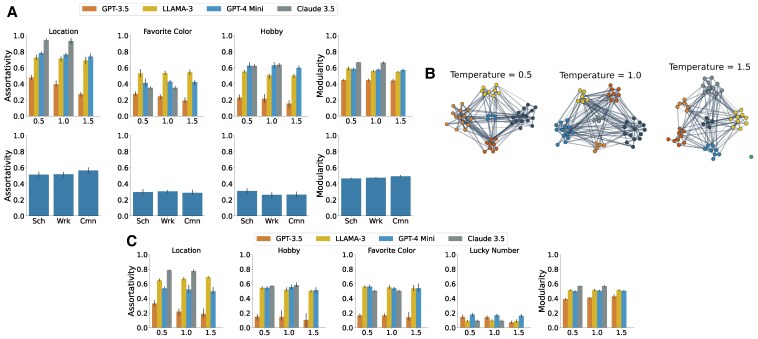
Results for principle 3 (Homophily) and principle 4 (Community structure due to homophily). A) Assortativity and Louvain modularity by principle 3 (n=50, five runs per row) across school, work, and community settings. All comparisons to the random null model (R=0) are statistically significant (P<0.0003, *t*-tests with Bonferroni correction for three tests). Modularity is also significantly greater than 0 (P<0.001). B) Network examples and communities for GPT-3.5 agents. Compared to a null model where agents connect randomly (R=0). C) Influence of distractor features (favorite color and lucky number) on homophily. Compared to a random null model with R=0. All results are statistically significant (P<0.00025), with Bonferroni correction over three tests (location, favorite color, and hobby).

As shown in Fig. 3, LLMs consistently yield positive assortativity across all features and models, with results highly significant (P<0.0003, *t*-tests with Bonferroni correction). To probe feature salience, we introduced a distractor attribute (lucky number 0–9). This feature showed much lower assortativity, though still statistically significant (P<0.00025, *t*-tests with Bonferroni correction), confirming that it is less influential in link formation. By contrast, favorite color produced homophily levels comparable to hobbies, echoing minimal group theory (39): even arbitrary distinctions can drive in-group favoritism.

Overall, these findings show that LLMs not only capture strong homophily effects but also reproduce subtle human-like behaviors, forming links around both meaningful and trivial shared traits. This underscores their potential as tools for social simulation while raising concerns about bias and fairness in networked AI systems.

### Macro-level principles

#### Principle 4: community structure

The community structure of networks refers to the organization of nodes or individuals within a network into distinct and densely interconnected groups or clusters (33, 40–42). Identifying community structures is crucial for understanding the overall dynamics of a network, as it reveals patterns of relationships and interactions that might not be apparent at the global level.

Both triadic closure and homophily contribute to the formation of community structures. By examining how these two factors contribute to network formation, we aim to gain insights into the underlying mechanisms driving community dynamics in LLM-generated networks. We employ the simulation results presented in the synthetic networks to determine whether community structure in networks generated by LLMs emerges from triadic closure or homophily.

First, we consider the networks generated in Fig. 2. We examine how LLM agents’ choices strengthen the network’s community structure. Specifically, we leverage the fact that the SBM graph has a pre-existing community structure and measure how the newly formed links reinforce this structure. Visual inspection reveals that the newly added links, represented by the bold edges, primarily occur within each cluster, thereby reinforcing the community structure.

To measure the emergence of communities in the triadic closure case, we initially examine the probability of forming an edge within the same community ($\hat{\;p}$). The quantity $\hat{p}$ is calculated by the ratio of edges in $G_{T}\setminus G_{1}$ (newly formed edges) connecting nodes within the same cluster:


$$\hat{p}=\frac{\left|\left\{\{i,j\}\in E(G_{T})\setminus E(G_{1})\;:\;y_{i}=y_{j}\right\}\right|}{\left|E(G_{T})\setminus E(G_{1})\right|},$$

where $y_{i},y_{j}\in A,B$ denote the community memberships of nodes *i* and *j*, respectively. A value of $\hat{\;p}$ exceeding 0.5 suggests a triadic closure tendency and community structure.

Figure 2 shows that $\hat{p}$ is significantly higher than 0.5 (P<0.001, *t*-test compared to 0.5) and is significantly bigger compared to a random null model where connections are made at random. All in all, this indicates that most edges are within the same community, strengthening the community structure.

Next, we investigate the community structure resulting from homophily using modularity maximization (40). Modularity quantifies the discrepancy between the actual number of edges within communities and the expected number in a random network with identical node count and degree distribution, following the Chung-Lu model (43). This model assumes that nodes maintain their weighted degree, with edges distributed randomly. The weighted modularity *Q* (41) for a graph with edge weights $w_{ij}$ and *C* communities is defined as


$$Q=\sum_{c=1}^{C}\left[\frac{L_{c}}{W}-r{\left(\frac{k_{c}}{2W}\right)}^{2}\right].$$

Here, *W* represents the total edge weights, $L_{c}$ the intra-community link weights for community *c*, $k_{c}$ the total weighted degree within community *c*, and *r* the resolution parameter, set to 1 for our analysis. High modularity values (e.g. greater than 0.5) indicate significant community structuring, diverging from the random model.

Firstly, we note that when the experimental setting for principle 2 (cf. Fig. 2) is initialized with an SBM or an Erdös–Renyi network, we obtain positive modularity Q>0 (P<0.001; *t*-test comparing with 0).

Secondly, regarding the homophily experiment (cf. Fig. 3), for the network’s weights, we use the number of common attributes shared between each pair of nodes: $w_{ij}=|\{k\;:\;x_{i}^{\left(k\right)}=x_{\;j}^{\left(k\right)}\}|$ for each link (i,j) in the final network. Here, $x_{i}^{\left(k\right)}$ and $x_{j}^{\left(k\right)}$ correspond to the *k*th features of $x_{i}$ and $x_{j}$, respectively.

In Fig. 3, various colors represent the communities identified by the Louvain algorithm at different temperatures for GPT-3.5. Notably, communities appear more distinct at lower temperatures, likely due to reduced randomness in decision-making at these temperatures.

Figure 3 presents the distribution of Louvain modularity values across simulations across different LLM models and different environments, indicating consistent community structure with positive modularity at all temperatures, confirmed by a *t*-test against a modularity of Q=0 for a random graph (P<0.001).

Our results demonstrate that community structures manifest in networks generated by LLMs, driven by both triadic closure and homophily.

#### Principle 5: small-world

The small-world phenomenon is characterized by networks where nodes are interconnected in tight clusters. Yet, the average distance between any two nodes remains relatively short, typically scaling logarithmically with the network size (34, 35). This balance between high clustering and short path lengths characterizes small-world networks.

A small-world network is defined by its *average shortest path length *L**, which grows logarithmically with the size of the network *n*, expressed as L∼log(n). Our analysis utilizes the Watts–Strogatz model (35) as a benchmark to investigate whether LLMs can generate networks exhibiting small-world characteristics. This model has a delicate balance between local clustering and short average path lengths: Nodes tend to form clusters or groups (triadic closure), exhibiting a high level of interconnectedness within these local neighborhoods, whereas at the same time, the existence of a few long-range connections ensures that the entire network is reachable with relatively few steps (13, 44, 45).

We employ a modified version of the model, where edge rewiring is informed by LLM queries, based on the current network structure. The generation process is parametrized by the number of nodes (*n*), average degree (*k*), and the rewiring probability (*β*). See details in the Materials and methods section.

We generated networks of various sizes, ranging from n=10 to n=100, to explore the relationship between the network size (*n*) and two key metrics: the average shortest path length (*L*) and the average clustering coefficient (*C*). For this analysis, we considered values of *β* set at 0.25, 0.5, and 0.75, with a fixed k=5 to serve as a consistent parameter.

**Fig. 4.**
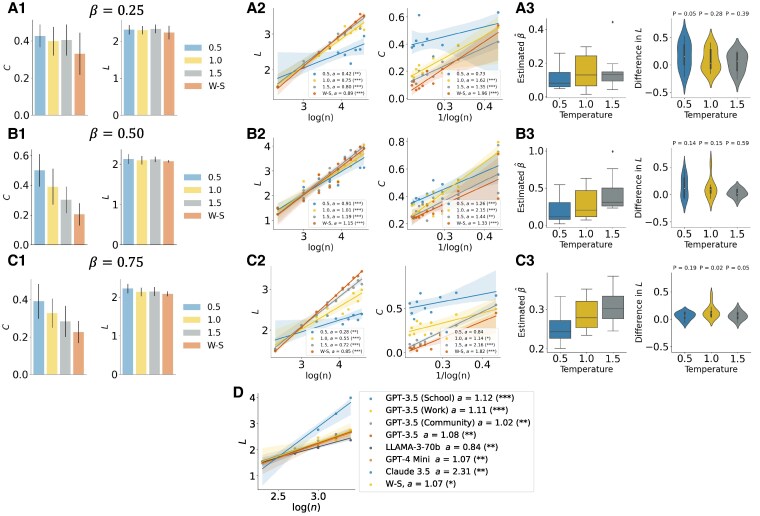
Fitted results for principle 5 (small world) for β=0.25,k=5 (A1–A3), β=0.5,k=5 (B1–B3), and β=0.75,k=5 (C1–C3). A1–C1) *Average clustering coefficient C* the *average shortest path length L*. The comparison is made with respect to a Watts–Strogatz graph with n=50,k=5,β∈{0.25,0.5,0.75}. The error bars correspond to 95% CI. The results are compared against the Watts–Strogatz model with the same parameters *k* and *β* as a null model. The *t*-test comparing *L* and *C* for the LLM-generated networks and Watts–Strogatz networks yields P>0.05 (Bonferroni correction for two tests). A2–C2) Regression plots relating *average shortest path length* (*L*) and *average clustering coefficient* (*C*) with *n*. The value *a* in legends represents the effect size (slope of the regression lines). A3–C3) Estimated values $\hat{\beta }$ of β∈{0.25,0.5,0.75} for LLM-generated networks based on matching the average clustering coefficient and difference in the average shortest path between LLM-generated networks and Watts–Strogatz with the estimated rewiring probability $\hat{\beta }$ for GPT-3.5 agents. We report the *P*-values of the *t*-test comparing the average shortest path length of the LLM-generated networks and the average shortest path length of the Watts–Strogatz graphs with rewiring probability $\hat{\beta }$. D) Regression plot for the relation L∼log(n) for different LLM models and environments (school, work, community) for β=0.25 and k=5. The legend shows the effect size (*a*) and the *P*-value. The results are compared against the Watts–Strogatz model with the same parameters *k* and *β* as a null model. (*: P<0.025; **: P<0.005, and ***: P<0.0005, Bonferroni correction for two tests; *L* and *C*.).

However, when directly compared with the Watts–Strogatz model, the networks generated by the LLMs do not precisely replicate the characteristics of Watts–Strogatz networks for the corresponding rewiring probabilities (*β*). As illustrated in panels (a1–c1) of Fig. 4, we fail to reject the null hypothesis at level 0.05 that the LLM-generated networks have the same average shortest path length as the Watts–Strogatz model for the rewiring probabilities (*β*) of 0.25, 0.5, and 0.75 (*t*-test comparing the average shortest path lengths, Bonferroni correction for two tests). Additionally, LLM-generated networks also fail to reject the null hypothesis at a level of 0.05 that the LLM-generated networks have the same average clustering coefficient as the Watts–Strogatz model for the same rewiring probabilities *β* (using the Bonferroni correction for two tests). These results may suggest similarities in the network structure and connectivity patterns between the LLM-generated networks and the classical Watts–Strogatz model.

We also provide regression analysis by examining the correlation between the average shortest path length and average clustering coefficient versus log(n) (refer to Fig. 4). We found that across all tested temperatures, the relationships were statistically significant, with most regressions yielding statistically significant results after applying Bonferroni correction for two tests (P<0.0005). This indicates that the average shortest path length increases proportionally with log(n). Similarly, for the average clustering coefficient, we demonstrated that it inversely scales with 1/log(n), with the majority of regression analyses also showing high statistical significance after Bonferroni correction for the two tests (P<0.0005). These findings align with the small-world properties of organizational networks as documented in the study by Ref. (46), suggesting that these characteristics are not only prevalent but also predictable across different network sizes. To quantify how LLM-generated networks resemble Watts–Strogatz networks, we fit the estimated $\hat{\beta }$ values for each LLM-generated network. In Fig. 4, we plot the estimated values for $\hat{\beta }$ for each value of *β* and each temperature. Here, *P*-values result from a *t*-test comparing with the average shortest path length of Watts–Strogatz with rewiring probability $\hat{\beta }$. These results show that while the average shortest path lengths are not identical, they are sufficiently close, with the differences not being statistically significant at the 0.1 level for most temperature settings. Finally, as Fig. 4 shows, the relation L∼log(n) holds for different LLM models and environments.

In conclusion, our analysis demonstrates that LLM-generated networks exhibit key small-world properties, with logarithmic scaling of average shortest path lengths and inverse logarithmic scaling of average clustering coefficients. While these networks do not perfectly align with the Watts–Strogatz model, they exhibit similar structural characteristics.

### Decisions on real-world networks with heterogeneous agents

#### Real-world datasets

To study LLM behavior in real-world contexts, we use four datasets spanning two domains. From the *Facebook100* collection (47), we analyze three college friendship networks. We also include two networks from (23): the *Andorra* telecommunication dataset, which records nationwide mutual calls with caller/callee attributes, and the *MobileD* employment dataset, where links represent calls or texts among managers and subordinates. All datasets provide agents with heterogeneous profiles, whose statistics are reported in the SI Appendix.

To infer network formation tendencies, we adopt a discrete choice modeling framework (48, 49), treating each link decision as a sequential choice from a candidate set of alternatives (see Materials and methods).

#### Candidate set construction for network decisions

At each decision step *t*, a query node $i_{t}$ selects a link from a set of candidate nodes $A_{t}$ with size $|A_{t}|=A$. Given the limited context window of LLMs, we consider two alternative strategies for constructing the candidate set $A_{t}$:

#### Uniform sampling

We uniformly sample *A* nonneighbor nodes from the graph. This approach serves as a neutral baseline, ensuring that the alternatives presented to the model are selected without structural or feature-based bias. Uniform sampling reflects a scenario where the agent has no a priori ranking or filtering of candidates and evaluates all choices purely based on the features provided in the prompt.

#### Recommendation-based sampling

We also consider a more realistic and structured candidate selection method that mimics the behavior of recommender systems. In this approach, we use a supervised link prediction model based on logistic regression to compute the likelihood of a link between each candidate pair (u,v) (cf. (50)). The model takes as input common structural features known to be predictive of link formation, such as similarity, the number of common neighbors between *u* and *v*, the preferential attachment score between *u* and *v*, the Jaccard similarity between the neighborhoods of *u* and *v*, and the Adamic-Adar index (see the Materials and methods for a description of the recommendation system and the SI Appendix for the effect sizes and AUC of the recommendation system). We then select the top-*A* highest-scoring nodes as the candidate set $A_{t}$ for each query node $i_{t}$. This method mirrors how real-world systems (e.g. social media friend suggestions, hiring portals, content feeds) narrow down decision spaces through algorithmic filtering based on network and user features.

#### Effects for uniform sampling

Regression results (see SI Appendix) show that homophily is the dominant driver of LLM link formation. Across all datasets and models, homophily coefficients (${\hat{\theta }}_{H}$) are the largest and highly significant (P<0.05), often strongly so—for example, in Caltech36, GPT-3.5, GPT-4, and Llama 3 70b Instruct yield values of 0.65, 1.95, and 2.43 (P<0.001). This indicates that, like humans, LLMs prioritize similarity when forming connections. Also, in the company context (MobileD), we find that although homophily has the largest magnitude, the coefficient is negative, indicating a strong (but dominant) effect. By examining the reasoning of the agents, we find that the agents exhibit “career-advancement dynamics” as subordinates prefer to network with managers.

Preferential attachment plays a secondary role. Coefficients (${\hat{\theta }}_{\mathrm{PA}}$) are generally positive and significant, though smaller than for homophily—for instance, 0.19 and 0.39 for GPT-3.5 and Llama 3 70b Instruct on Swarthmore42 (P<0.001). Thus, LLMs consider the degree but less strongly than similarity.

Triadic closure effects (${\hat{\theta }}_{\mathrm{TC}}$) are more variable: typically positive and significant, but sometimes negative, as in GPT-3.5 on Andorra (−0.24, P<0.05), reflecting dependence on network structure (e.g. low clustering in Andorra). Results are robust to larger candidate sets (A∈{50,100}; see SI).

Overall, while all three principles shape behavior, homophily is consistently the most influential.

#### Effects for recommendation-based sampling

Our analysis reveals that the key behavioral patterns of LLM agents, namely, the relative strength of their preferences for homophily, triadic closure, and preferential attachment, are largely consistent across different candidate selection strategies. Specifically, we observe high Spearman correlations and low TV distances in the majority of comparisons, both when using the same sampling strategy and when comparing the uniform sampling method to the recommendation-based approach. Homophily remains the dominant factor in the agent’s decisions and is context-dependent even when the agents interact with the recommendation system.

These results suggest that the observed LLM behaviors are, in the majority, robust to variations in how the candidate set $A_{t}$ is constructed.

#### Average marginal effects

Average marginal effects (AMEs), reported in the SI Appendix, complement the regression results by quantifying the change in choice probability from a one-unit change in each feature. Homophily shows the largest AMEs across datasets, often exceeding 1.0 and reaching 2.5 under recommendation-based sampling (e.g. GPT-4 Mini on UChicago30). Preferential attachment effects are smaller but consistently positive, while triadic closure varies—positive in several Facebook100 networks, but often negative in Andorra and MobileD, suggesting cross-community links. Recommendation-based sampling amplifies the dominant mechanism (typically homophily in Facebook100 and preferential attachment in MobileD) while preserving the overall ranking of principles.

#### Change of graph statistics

Adding a small fraction of new edges (≤5%) leaves global network properties—degree distribution, spectrum, and component sizes—largely unchanged. The main shifts occur locally in clustering, especially under Uniform sampling, while the recommendation-based strategy induces even fewer changes. These patterns are consistent across temperature settings, indicating that LLM-driven link formation preserves overall network structure while shaping local connectivity.

### Human baseline

#### Survey.

To assess how closely the network formation preferences of LLM agents align with human decision-making, we conducted a controlled survey-based experiment involving both human participants and LLMs. The experiment was designed to elicit link formation choices in two distinct social contexts: (i) a social network where participants assumed the role of a student forming friendships within a college social network and (ii) a company network where participants assumed the role of an employee making professional connections within a company network.

For each scenario, the participant was presented with a focal node (representing themselves) and A=3 candidate profiles containing a value indicating similarity as well as relevant network statistics (degree, common neighbors) with the focal profile. The participant was then asked to select exactly one candidate with whom to form a connection and rate the criteria—among similarity, degree, and common neighbors—that they considered when choosing the specific profile. The Materials and methods section contains more information about the experimental setup.

We recruited human participants via the Prolific platform, ensuring diversity in demographic backgrounds. We obtain n=100 and n=103 responses for the social and company network contexts, respectively. To compare with LLM models, we administered the same dynamically generated survey inputs that we provided to each participant to an LLM—preserving the wording, structure, and candidate attributes—for the five LLM models.

This experimental design enables a direct, context-controlled comparison between human and LLM decision-making patterns. By presenting an identical set of alternatives, we can measure alignment in two complementary ways: (i) at the *principle level*, by estimating discrete choice models for both humans and LLMs and comparing the inferred effect sizes for homophily, triadic closure, and preferential attachment and (ii) at the *choice level*, by comparing the Borda count vectors between how the participants ranked each criterion in their choices. Furthermore, the two scenarios are chosen to reflect socially and professionally relevant settings, as is the case with real-world datasets.

#### LLM-human alignment in network formation contexts

**Fig. 5.**
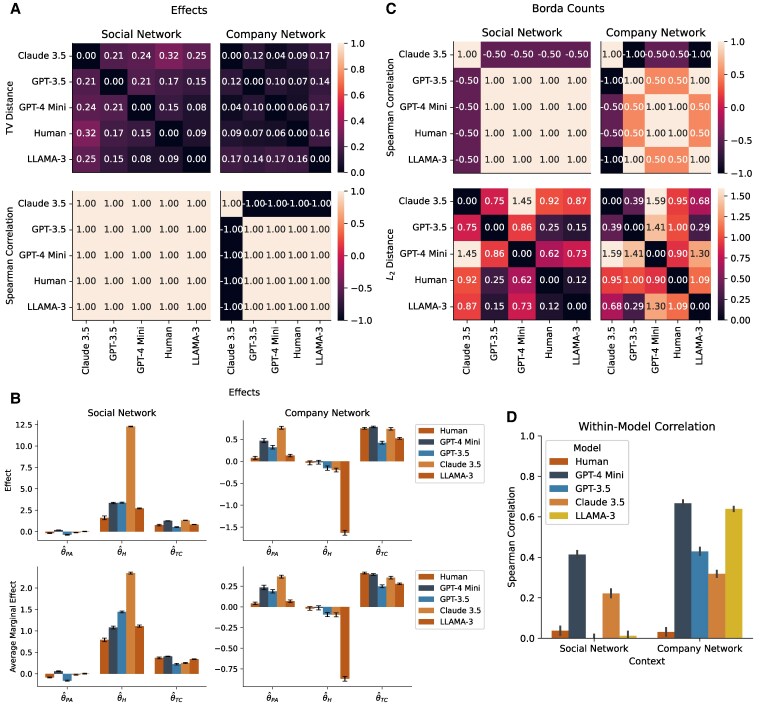
Measurement of LLM-Human alignment for two contexts: Social network (n=100), and company network (n=103). A) TV distance between fitted models and Spearman correlation between estimated effects. B) Effects and average marginal effects with standard errors to measure alignment between how different models and humans rank preferential attachment, homophily, and triadic closure. C) Spearman correlation and $L_{2}$ distance between the average Borda count vectors to measure alignment between how different models and humans rank preferential attachment, homophily, and triadic closure. D) Within-model Spearman correlations between the decisions within each model to measure alignment within the decisions of one model.

Figure 5 summarizes the results of our alignment study. Across all scenarios, the rankings of network formation principles based on effect sizes are perfectly correlated between humans and LLMs, and the Borda vector averages are likewise almost perfectly correlated (except Claude 3.5). The total variation (TV) distances between the discrete choice models inferred from human and LLM choices are consistently small—below 0.32 in all cases and typically below 0.10—indicating high distributional similarity (cf. Fig. 5A–C). In the vast majority of cases, the signs of the estimated effects also match between humans and LLMs. Consistent with our earlier observations, we find strong homophily in the social network context (${\hat{\theta }}_{H}>0;P<0.001$) and strong heterophily in the company network context (${\hat{\theta }}_{H}<0;P<0.001$). Additionally, the ranking of the effects is consistent with our findings on measuring the regression coefficients in real-world networks (cf. SI Appendix).

While aggregate-level alignment between human and LLM decisions is high across models (cf. Fig. 5A–C), notable differences emerge in *within-model* agreement. Specifically, LLMs exhibit substantially higher internal consistency in their decision rankings than humans, who display greater variability in their within-model rankings (cf. Fig. 5D).

## Discussion

### Summary of findings and managerial insights

Our study demonstrates that LLM-generated networks accurately reproduce well-established microlevel principles of human behavior, including homophily, triadic closure, and preferential attachment, as well as macrolevel structures such as community formation and small-world properties. Discrete choice modeling confirms that LLMs prioritize homophily, followed by triadic closure and preferential attachment. This suggests that in social or organizational settings, LLMs may mirror human tendencies, with implications for practice: they can inadvertently reinforce echo chambers, hierarchies, or information silos, but also enable realistic simulations for testing interventions or designing fairer information systems. Introducing a human baseline further validates the use of LLM agents as credible proxies in social network experiments, offering managers and researchers a cost-effective alternative to human-subject studies.

### Limitations and boundary conditions

LLMs exhibit biases that vary across models and contexts. For example, GPT-4 and Claude 3.5 show stronger preferences for high-degree nodes, while GPT-3.5 and LLaMA 3 display weaker versions of this tendency. Homophily is dominant in friendship networks, yet heterophily emerges in workplace settings, reflecting career dynamics. Moreover, LLM decisions are more correlated with one another than with humans, raising concerns about diversity and robustness. Thus, deploying LLMs in practice requires careful monitoring to avoid amplifying structural biases.

### Future research directions

Promising directions include studying LLMs in richer interactive settings such as dialog, embedding them in real platforms to counteract echo chambers or facilitate inclusive deliberation, and developing stronger structural comparisons between synthetic and empirical networks. LLMs can also provide realistic, privacy-preserving benchmarks for graph learning, expanding their role as tools for both scientific inquiry and applied network design.

## Materials and methods

### Experimental procedure

#### Experimental setup

We simulate network formation over *T* steps with networks $G_{1},G_{2},\ldots ,G_{T}$. The initial seed network $G_{1}$ evolves as, at each step *t*, a query node $i_{t}$ (either new or existing) selects up to *δ* links from a candidate set $A_{t}$. The LLM receives the attributes of these candidates, $F(A_{t})$ (e.g. node degrees, neighbors, common connections, community labels), formatted in JSON, and returns its link choices via a query call $Q(A_{t},i_{t},\delta )$. Following a zero-shot design, no examples are provided, allowing us to observe the models’ innate preferences.

To account for stochasticity, we run experiments under multiple temperature settings. For all models except Claude 3.5, we use 0.5, 1.0, and 1.5; for Claude 3.5, we use 0.5 and 1.0.

### Details for small-world experiments

We modify the Watts–Strogatz model by introducing LLM-guided rewiring. As in the classical model, we begin with a ring lattice of *n* nodes in which each node is connected to k/2 neighbors on the left and k/2 neighbors on the right. To generate $G_{t}$, we then consider the k/2 rightmost neighbors of each node and rewire each edge with probability *β*. Unlike the original Watts–Strogatz model, where rewiring is performed uniformly at random, here each rewiring decision is determined by querying an LLM. The model is provided with the full network structure, including all nodes and their neighborhoods, and the LLM selects the new endpoint of the rewired edge. This modification preserves the small-world foundation of the Watts–Strogatz model while replacing random rewiring with LLM-driven choices.

### Real-world network experiments

For each focal node $i_{t}$ at time *t*, we randomly removed one of its existing neighbors to construct a seed network $G_{1}$ for the LLM agents. During link formation, $i_{t}$ was then presented with a candidate set $A_{t}$ consisting of the removed friend and other nonneighbors, along with their attributes and the current network structure. The LLM was asked to select one candidate to connect with, and this process was repeated sequentially across nodes.

We model these choices using a multinomial logit (MNL) framework. The utility of connecting to node $j\in A_{t}$ is


$$U_{ij,t}=\theta_{\mathrm{PA}}\log\;d_{\;j,t}+\theta_{H}\log\;w_{ij}+\theta_{\mathrm{TC}}\log\;c_{ij,t}+\epsilon_{ij,t},$$

where $d_{\;j,t}$ is the degree of *j* (preferential attachment), $w_{ij}$ measures attribute similarity (homophily), and $c_{ij,t}$ counts common neighbors (triadic closure). The error term $\epsilon_{ij,t}$ follows an i.i.d. Gumbel distribution. The resulting MNL model yields probabilities


$$p_{ij,t}=\frac{d_{\;j,t}^{\theta_{\mathrm{PA}}}w_{ij}^{\theta_{H}}c_{ij,t}^{\theta_{\mathrm{TC}}}}{\sum_{r\in A_{t}}d_{r,t}^{\theta_{\mathrm{PA}}}w_{ir}^{\theta_{H}}c_{ir,t}^{\theta_{\mathrm{TC}}}},$$

and the parameters $\theta_{\mathrm{PA}},\theta_{H},\theta_{\mathrm{TC}}$ are estimated by maximum likelihood. Standard errors and *p*-values are obtained following (49), with optimization via L-BFGS-B (51, 52). Full derivations and robustness checks are provided in the SI Appendix.

#### Candidate set construction for network decisions

At each decision step, a focal node must choose a new link from a set of candidates. Because LLMs have limited context windows, we consider two strategies for building these candidate sets: As a neutral baseline, we randomly draw a fixed number of nonneighbor nodes. This ensures that the options presented to the model are unbiased and evaluated solely based on the features shown in the prompt. Also, as a more structured alternative, we train a logistic regression model on the initial network (before any LLM decisions) to estimate how likely two nodes are to form a link. The model uses standard link-prediction features such as similarity, common neighbors, and degree-based measures. At each step, we then select the top-scoring nodes for the focal node to form the candidate set. Further parameter details are provided in the SI Appendix.

#### Measuring alignment between models

For two LLM models M and $M^{\prime }$, we fit two discrete choice models ${\hat{\theta }}^{M}$ and ${\hat{\theta }}^{M^{\prime }}$, respectively and measure the following:

First, we measure $\mathrm{Spearman}({\hat{\theta }}^{M},{\hat{\theta }}^{M^{\prime }})$, which measures the difference in how the different LLM models rank preferential attachment, homophily, and triadic closure.

Second, we measure the total variation distance between the probabilities that each LLM model assigns to nodes parametrized by the MNL model. Specifically, the TV distance is calculated by sampling a time index *t* uniformly in {1,…,T} and a pair of a node $u_{t}$ and an alternative set $A_{t}$ from $G_{t}$ as $d_{TV}(M,M^{\prime })$  $=\frac{1}{2}E_{t∼U[T],(u_{t},A_{t})∼G_{t}}\left[\sum_{v\in A_{t}}|\frac{d_{v,t}^{{\hat{\theta }}_{\mathrm{PA}}^{M}}w_{uv}^{{\hat{\theta }}_{H}^{M}}c_{uv,t}^{{\hat{\theta }}_{\mathrm{TC}}^{M}}}{\sum_{r\in A}d_{r,t}^{{\hat{\theta }}_{\mathrm{PA}}^{M}}w_{ur}^{{\hat{\theta }}_{H}^{M}}c_{ur,t}^{{\hat{\theta }}_{\mathrm{TC}}^{M}}}-\frac{d_{u,t}^{{\hat{\theta }}_{\mathrm{PA}}^{j}}w_{uv}^{{\hat{\theta }}_{H}^{M^{\prime }}}c_{uv,t}^{{\hat{\theta }}_{\mathrm{TC}}^{M^{\prime }}}}{\sum_{r\in A}d_{r,t}^{{\hat{\theta }}_{\mathrm{PA}}^{M^{\prime }}}w_{uv}^{{\hat{\theta }}_{H}^{M^{\prime }}}c_{uv,t}^{{\hat{\theta }}_{\mathrm{TC}}^{M^{\prime }}}}|\right].$ In our implementation, we report the Monte-Carlo estimate.

### Human baseline

We ran a survey on Prolific with US-based adults (n=100 in the social network context, n=103 in the company context). Each participant was shown three alternatives described by: (i) the number of friends (degree), (ii) the number of common friends, and (iii) similarity. In the social network setting, similarity ranged from 0 to 10 to capture shared interests; in the company setting, it was binary, indicating whether the person was a coworker or a manager. Participants were asked to take the role of a college student (social network) or a nonmanagerial employee (company) and to rank the three attributes from least to most important, with an option to add qualitative reasoning. Standard Prolific sampling settings were used and duplicate submissions were blocked.

The study protocol was approved by the Institutional Review Board of Cornell University (Protocol #IRB0150009) and deemed exempt. Written informed consent was obtained from all participants prior to participation in the study.

To evaluate alignment between humans and models, we translated each participant’s ranking into a simple “Borda count” vector reflecting the order of importance they assigned to the three features. For models, we generated the same kind of vectors based on their decisions. We then compared humans and models in two ways: first, by looking at which principles (degree, common friends, and similarity) they emphasized most strongly; and second, by comparing the overall rankings they produced. Agreement was measured using correlation and distance metrics, and we also assessed consistency within each group by averaging how similar participants were to one another.

## Supplementary Material

pgaf317_Supplementary_Data

## Data Availability

The code and data used in this article are available at https://doi.org/10.5281/zenodo.16969696, and https://doi.org/10.5281/zenodo.17196412. The real-world social network data were obtained from Refs. (47) and (23).

## Previous Presentation

These results were previously presented at the Conference on Network Science and Economics, the SCECR conference, the IC2S2 conference, IBM Research, the INFORMS 2024 Annual Meeting, Cornell University, UIUC, and ASU. The authors would like to thank all the participants for their valuable feedback.

## Preprints

A preprint is available at https://arxiv.org/pdf/2402.10659.
