# Supplementary material for: Network formation and dynamics among multi-LLMs
Source: PNAS Nexus. 2025 Dec 2;4(12):pgaf317. doi: 10.1093/pnasnexus/pgaf317 (PMC12670380; doi:10.1093/pnasnexus/pgaf317)
Supplement: pgaf317_Supplementary_Data [file pgaf317_supplementary_data.pdf]

SI Appendix: Network Formation and Dynamics among Multi-LLMs*

Marios Papachristou[†] Yuan Yuan[‡]

This version: September 30, 2025

A Prompts

A.1 Network Formation Prompts

The general prompt we use is given in Algorithm 2. An example of this prompt is given at Algorithm 1.

*Corresponding author: Marios Papachristou (mpapachr@asu.edu).
†Department of Information Systems, W.P. Carey School of Business, Arizona State University, Tempe, AZ, USA.
‡Graduate School of Management, University of California Davis, Davis, CA, USA and OpenAI, San Fransisco, CA, USA.

Algorithm 1 Example prompt regarding social network data.

```
# Task
You are located in a school. Your task is to select a set of people to be friends
 with.

# Profile
Your profile is given below after chevrons:
<PROFILE>
    {
        "name" : "Person 0",
        "favorite subject" : "Chemistry",
        "neighbors" : ["Person 3", "Person 432", "Person 4", "Person 3", "Person
         32"]
    }
</PROFILE>

# Candidate Profiles
The candidate profiles to be friends with are given below after chevrons:

<PROFILES>
[
    {
        "name" : "Person 1",
        "favorite subject" : "Mathematics",
        "neighbors" : ["Person 3", "Person 4", "Person 23", "Person 65"]
    },
    {
        "name" : "Person 33",
        "favorite subject" : "History",
        "neighbors" : ["Person 342", "Person 2", "Person 12"]
    }, ...
]

</PROFILES>

# Output
The output should be given a list of JSON objects with the following structure

[
    {{
        "name" : name of the person you selected,
        "reason" : reason for selecting the person
    }}, ...
]

# Notes
- The output must be a list of JSON objects ranked in the order of preference.
- You can make at most 1 selection.
```

Algorithm 2 General Prompt used to implement $\mathcal{Q}(A_t, i_t, \delta)$.

```
# Task
Your task is to select a set of people to be friends with.

# Profile
Your profile is given below after chevrons:
<PROFILE>F({i_t})</PROFILE>

# Candidate Profiles
The candidate profiles to be friends with are given below after chevrons:

<PROFILES>F(A_t)</PROFILES>

# Output
The output should be given a list of JSON objects with the following structure

[
    {{
        "name" : name of the person you selected,
        "reason" : reason for selecting the person
    }}, ...
]

# Notes
- The output must be a list of JSON objects ranked in the order of preference.
- You can make at most δ selections.
```

Feature Representations for Prompts

Below, we give examples of the features used in the prompt presented in Algorithm 2. The features are formatted as a list of JSON objects, which are provided to the prompt.

Principle 1: Preferential Attachment. We have the following features:

```
[
    {
        "name" : 0,
        "neighbors" : [5, 7, 1, 6]
    },
    ...
]
```

Principle 2: Triadic Closure. We have the following features:

```
[
    {
        "name" : 0,
        "common_neighbors" : [5, 7, 1, 6]
    },
    ...
]
```

Principle 3: Homophily. We have the following features:

```
[
    {
        "name" : 0,
        "favorite_color" : "red",
        "hobby" : "hiking",
        "location" : "Boston"
    },
```

```
    ...
]
```

Principle 5: Small-World. We have the following features:

```
[
    {
        "name" : 0,
        "neighbors" : [5, 7, 1, 6]
    },
    ...
]
```

Real-World Data. We have the following features:

```
[
    {
        "name" : 0,
        "status" : "student",
        "major" : 10,
        "second major" : 93,
        "accommodation" : "house",
        "high_school" : 5,
        "graduation_year" : 2008
    },
    ...
]
```

We note that the initial Facebook100 dataset included gender information as a feature. We chose not to include gender as one of the features, as it has been shown that language models exhibit gender bias [5, 2, 1]. An example of the prompt using real-world social network data is given at Algorithm 1.

A.2 Survey Prompts

We use the prompt described in Algorithm 3 for the survey data.

Algorithm 3 Prompt used to implement the survey. When the context ω is a social network, the `profile_text` variable is set to be `'You are an undergraduate student at a university. You are looking for friends to connect with on a social network.'`. When the context is the company network, the `profile_text` variable is set to `'You are an employee at a company. You are looking for colleagues to connect with on a company network.'`

```
# Task
Your task is to select a set of people to be friends with.

# Profile
{profile_text}

# Candidate Profiles
The candidate profiles to be friends with are given below after chevrons:

<PROFILES>F(A_{i,ω})</PROFILES>

# Output
The output should be as a JSON object with the following structure

{{
    "name" : name of the person you selected (integer format),
    "reason" : reason for selecting the person,
    "ranking_degree" : ranking of how much you based your decision on the degree of
     the person (1 = most important, 2 = average important, 3 = least important),
    "ranking_similarity" : ranking of how much you based your decision on the
     similarity of the person (1 = most important, 2 = average important, 3 = least
     important),
    "ranking_common_friends" : ranking of how much you based your decision on the
     number of common friends with the person (1 = most important, 2 = average
     important, 3 = least important)
}}

# Notes
* The output must be a single JSON object ranked in the order of preference.
* You can make at most 1 selection.
* Your output must be contained within the json markdown cue.
* Rankings must be mutually exclusive, i.e. you cannot have the same ranking for two
  different attributes.
```

B Real-World Datasets

B.1 Resulting Regression Coefficients and Alignment Between Models

Hyperparameters. For the three datasets from Facebook100 are Caltech36 ($n = 769$) Swarthmore42 ($n = 1,659$), and UChicago30 ($n = 6,591$), we set the number of alternatives to be $A = 15$ and randomly sampled from the existing network. For the UChicago30 dataset, we consider a randomly sampled subset of $N = 2,000$ nodes because of the limited context window of the LLM models. For Andorra ($n = 32,812$) and MobileD ($n = 1,982$), we set the number of alternatives to $A = 5$ and consider a randomly sampled subset of $N = 1,000$ nodes.

Model	Preferential Attachment ($\hat{\theta}_{PA}$)	Homophily ($\hat{\theta}_{H}$)	Triadic Closure ($\hat{\theta}_{TC}$)	Log Likelihood	AIC
Caltech36 ($n = 769$ nodes, $m = 33,312$ edges, $N = 769$ samples, $A = 15$ alternatives each)					
GPT-3.5 + Uniform	0.20*** (0.002)	0.65*** (0.005)	-0.06 (0.006)	-2,088.21	4,184.41
GPT-4o Mini + Uniform	0.34*** (0.006)	2.13*** (0.03)	0.44*** (0.02)	-1,201.27	2,410.55
Claude 3.5 + Uniform	0.46*** (0.005)	0.55*** (0.01)	0.55*** (0.007)	-1,748.19	3,504.38
Llama 3 70b + Uniform	0.28*** (0.006)	2.43*** (0.02)	0.84*** (0.01)	-809.57	1,627.15
GPT-3.5 + RecSys	0.15** (0.002)	0.08 (0.007)	-0.60*** (0.02)	-2,114.41	4,236.82
GPT-4o Mini + RecSys	0.21*** (0.004)	2.32*** (0.005)	0.33** (0.005)	-1,611.38	3,230.77
Claude 3.5 + RecSys	0.65*** (0.002)	1.86*** (0.01)	0.20 (0.01)	-1,852.96	3,713.91
Llama 3 70b + RecSys	0.23*** (0.003)	4.13*** (0.01)	0.68*** (0.01)	-919.15	1,846.30
Swarthmore42 ($n = 1,659$ nodes, $m = 122,100$ edges, $N = 1,659$ samples, $A = 15$ alternatives each)					
GPT-3.5 + Uniform	0.19*** (0.008)	0.47*** (0.01)	0.00 (0.009)	-4,484.45	8,976.90
GPT-4o Mini + Uniform	0.27*** (0.21)	2.22*** (0.78)	0.57*** (0.43)	-1,899.09	3,806.19
Claude 3.5 + Uniform	0.36*** (0.002)	0.75*** (0.006)	0.55*** (0.004)	-3,563.02	7,134.03
Llama 3 70b + Uniform	0.39*** (0.003)	2.31*** (0.005)	0.62*** (0.004)	-1,820.26	3,648.52
GPT-3.5 + RecSys	0.14** (0.001)	0.11 (0.002)	-0.08 (0.002)	-4,564.89	9,137.78
GPT-4o Mini + RecSys	0.33*** (0.007)	2.94*** (0.01)	0.45*** (0.006)	-2,723.78	5,455.57
Claude 3.5 + RecSys	1.26*** (0.004)	1.22*** (0.007)	0.95*** (0.006)	-2,281.61	4,571.22
LLama 3 70b + RecSys	0.09 (0.007)	2.58*** (0.02)	1.18*** (0.009)	-610.00	1,228.00
UChicago30 ($n = 6,951$ nodes, $m = 416,206$ edges, $N = 2,000$ samples, $A = 15$ alternatives each)					
GPT-3.5 + Uniform	0.22*** (0.001)	0.48*** (0.004)	-0.02 (0.0005)	-8,157.38	16,322.77
GPT-4o Mini + Uniform	0.27*** (0.005)	2.22*** (0.019)	0.57*** (0.011)	-1,899.09	3,806.19
Claude 3.5 + Uniform	0.43*** (0.003)	0.78*** (0.005)	0.39*** (0.002)	-6,604.77	13,217.54
Llama 3 70b + Uniform	0.43*** (0.007)	2.57*** (0.014)	0.32*** (0.005)	-3,689.00	7,386.00
GPT-3.5 + RecSys	0.14*** (0.002)	-0.08 (0.006)	0.18** (0.007)	-4,459.64	8,927.28
GPT-4o Mini + RecSys	0.32*** (0.001)	3.44*** (0.01)	-0.74*** (0.005)	-3,154.27	6,316.53
Claude 3.5 + RecSys	0.75*** (0.002)	1.68*** (0.005)	0.17* (0.003)	-2,386.04	4,780.09
LLama 3 70b + RecSys	0.27*** (0.004)	2.81*** (0.02)	0.53** (0.01)	-661.75	1,331.50
Andorra ($n = 32,812$ nodes, $m = 513,931$ edges, $N = 1,000$ samples, $A = 5$ alternatives each)					
GPT-3.5 + Uniform	0.53*** (0.001)	0.21* (0.01)	-0.24*** (0.002)	-1,712.91	3,433.83
GPT-4o Mini + Uniform	0.54*** (0.004)	3.47*** (0.06)	-0.09* (0.01)	-1,002.11	2,012.22
Claude 3.5 + Uniform	0.54*** (0.003)	1.94*** (0.009)	-0.15*** (0.003)	-1,541.77	3,091.55
Llama 3 70b + Uniform	0.38*** (0.003)	3.92*** (0.02)	-0.04 (0.01)	-985.95	1,979.91
GPT-3.5 + RecSys	0.31*** (0.03)	-0.07 (0.009)	-0.43*** (0.007)	-1,722.42	3,452.84
GPT-4o Mini + RecSys	0.11 (0.003)	3.68*** (0.008)	-0.64*** (0.006)	-938.74	1,885.47
Claude 3.5 + RecSys	0.38*** (0.003)	1.78*** (0.01)	-0.41*** (0.005)	-1,651.67	3,311.33
Llama 3 70b + RecSys	0.53*** (0.002)	3.63*** (0.01)	-0.12* (0.003)	-1,238.22	2,484.45
MobileD ($n = 1,982$ nodes, $m = 25,470$ edges, $N = 1,000$ samples, $A = 5$ alternatives each)					
GPT-3.5 + Uniform	1.06*** (0.003)	-0.94*** (0.009)	-0.02 (0.001)	-1,663.42	3,334.84
GPT-4o Mini + Uniform	1.38*** (0.02)	-0.85*** (0.02)	0.87*** (0.01)	-880.39	1,768.78
Claude 3.5 + Uniform	0.71*** (0.009)	-2.44*** (0.02)	1.13*** (0.005)	-1,197.92	2,403.83
Llama 3 70b + Uniform	1.04*** (0.005)	-0.36** (0.01)	0.71*** (0.002)	-1,269.42	2,546.83
GPT 3.5 + RecSys	1.68*** (0.006)	-0.35** (0.008)	-0.91*** (0.006)	-1,613.85	3,235.69
GPT-4o Mini + RecSys	3.16*** (0.01)	-0.49* (0.02)	0.67*** (0.01)	-681.39	1,370.78
Claude 3.5 + RecSys	1.85*** (0.01)	-0.87*** (0.006)	0.16** (0.007)	-1,542.90	3,093.80
Llama 3 70b + RecSys	1.43*** (0.01)	1.05*** (0.007)	0.36*** (0.005)	-1,468.61	2,945.22
Note: *: $P < 0.05$, **: $P < 0.01$, ***: $P < 0.001$					

Table 1: Effect sizes for real-world networks from Facebook100 [4], the Andorra dataset [7], and the MobileD dataset [7] for several LLMs for temperature set to 0.5. We test two sampling strategies: a *uniform* strategy where A_t is sampled uniformly from the set of nodes, and a *recommender system* (RecSys) based on logistic regression and trained on pairwise node similarities and network characteristics (number of common neighbors, Jaccard similarity, preferential attachment score, and the Adamic-Adar index). See Methods and Materials for more information on the sampling strategies. Average marginal effects (cf. SI Appendix B.3.3) show that homophily is the strongest driver of link formation, with recommendation-based sampling amplifying the dominant mechanism in each dataset while preserving the overall ranking of behavioral factors.

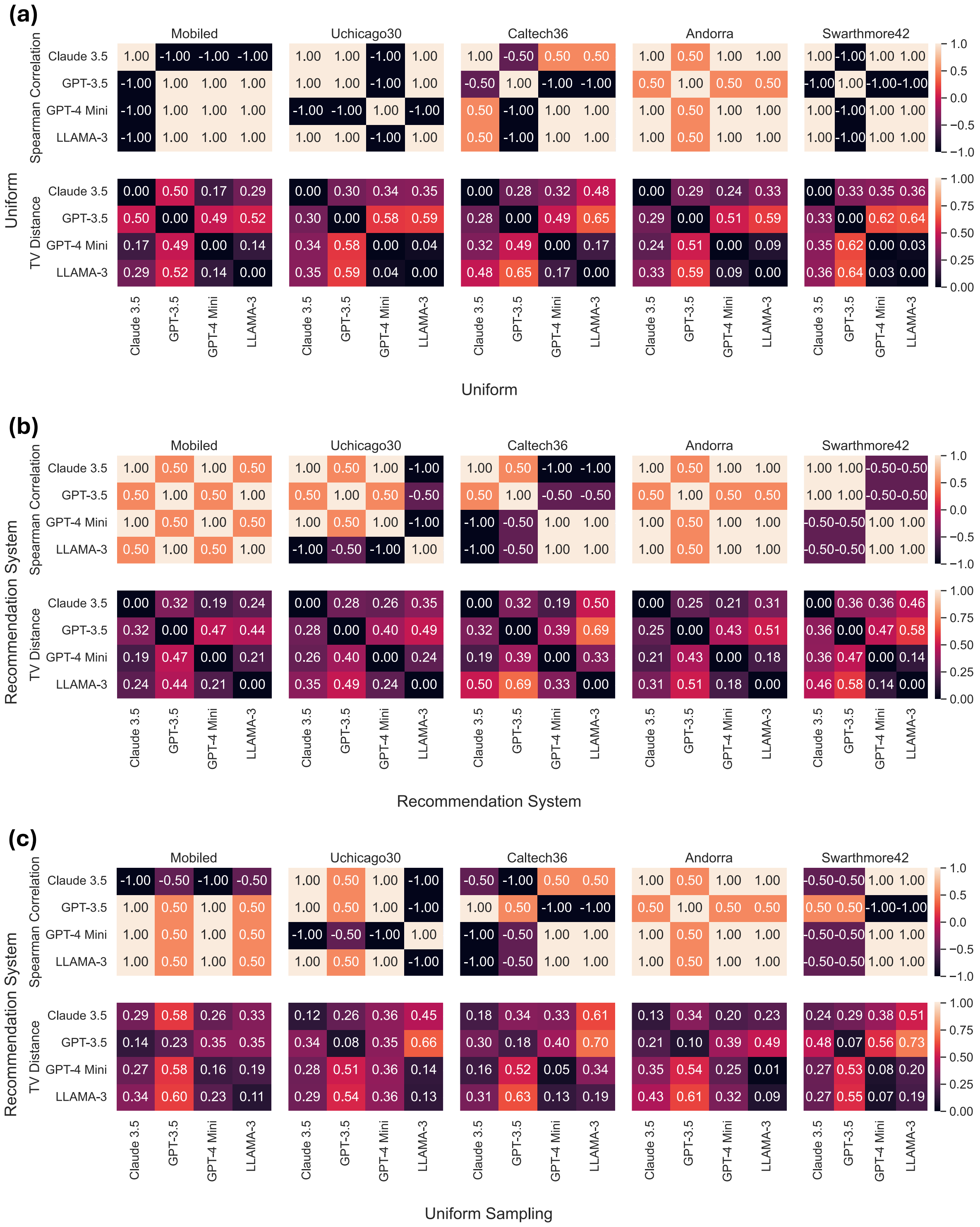

Figure 1: Comparison between the network formation decisions among different models for the uniform and the recommendation system sampling strategies. We report the Spearman correlation between the effects corresponding to the fits as well as the total variation (TV) distance between the corresponding fitted models.

B.2 Statistics of Real-World Datasets

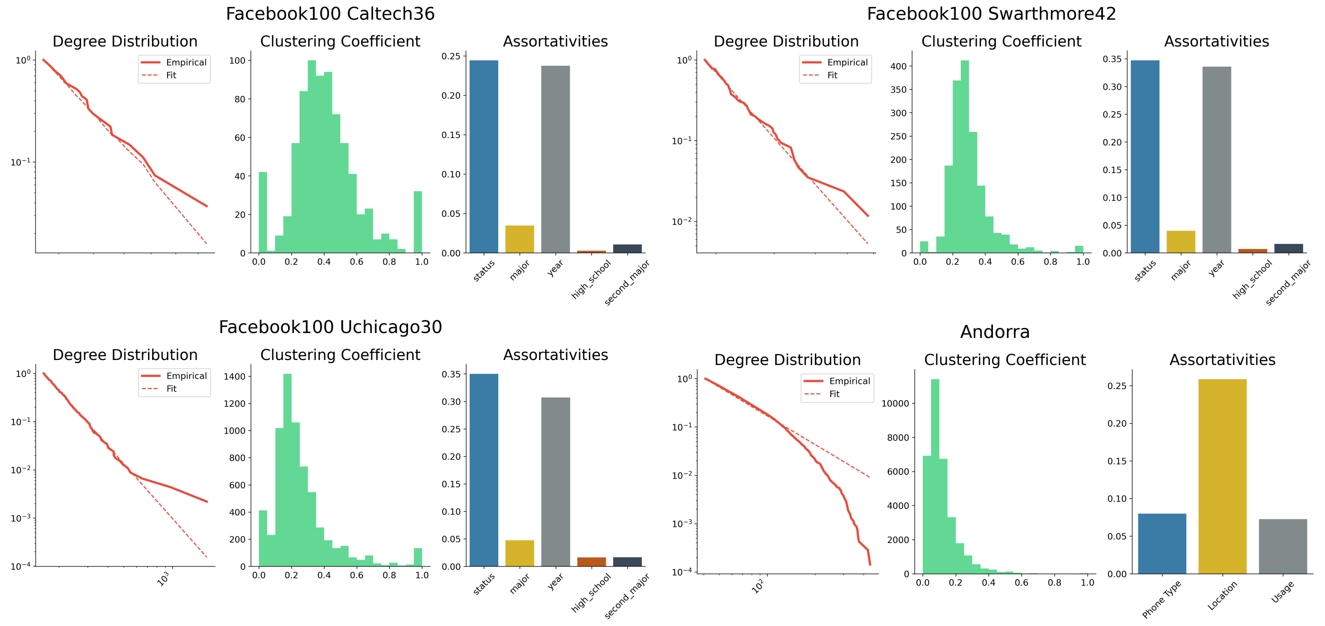

Figure 2: Distributions of real-world datasets analyzed in our study, including degree, clustering coefficients, and the assortativities of the attributes included in the datasets.

B.3 Robustness of Results to Sampling Strategies

B.3.1 Recommendation System Parameters

	Constant	Similarity	Common Neighbors	Jaccard	Adamic-Adar	PA Score	AUC Score
Caltech36	-2.9861***	0.5103***	-0.9384***	9.9513***	4.9751***	-4.28×10^{-5}***	95.2
Swarthmore42	-3.0713***	0.3323***	-0.8603***	29.0475***	4.5827***	-4.596×10^{-6}	94.8
UChicago30	-3.1467***	0.5493***	-1.5431***	59.5934***	8.3355***	3.575×10^{-5}***	97.05
Andorra	-2.1337***	0.2247***	-2.0713***	96.1618***	13.2652***	3.00×10^{-4}***	92.4
MobileD	-4.6000***	0.4075*	-1.9072***	24.7593**	14.6024***	-0.0058***	98.9
Note							* : $P < 0.05$, ** : $P < 0.01$, *** : $P < 0.001$

Table 2: Recommendation System Parameters.

Table 2 shows the effects and the AUC score for the recommender system based on logistic regression.

B.3.2 Change in Graph Statistics due to Different Sampling Strategies

Across all datasets and models, the Kolmogorov–Smirnov statistics indicate that adding a small fraction of new edges ($\leq 5\%$) produces only minor shifts in the degree distribution, spectrum, and sizes of connected components, with most p-values far above the 0.05 threshold. Significant changes arise primarily in the local clustering coefficient—particularly under the Uniform strategy—suggesting localized structural effects without major disruption to global network properties. In contrast, the Recommendation System strategy yields even fewer significant differences, with most metrics remaining statistically indistinguishable from the original graphs. These results indicate that, at this perturbation scale, LLM-driven edge additions preserve the overall network structure, with strategy choice influencing the extent of local structural change.

Name	Model	Degrees (KS)	P-value	Sizes of CCs (KS)	P-value	Spectrum (KS)	P-value	LCC (KS)	P-value	% New Edges
					Uniform					
Caltech36	GPT-4 Mini	0.05	0.3	0.1	1	0.06	0.2	0.04	0.6	5
Swarthmore42	GPT-4 Mini	0.02	0.8	0.2	1	0.02	1	0.02	1	3
UChicago30	GPT-4 Mini	0.02	0.3	0.06	1	0.02	0.2	0.006	1	1
Caltech36	GPT-3.5	0.06	0.1	0.5	0.6	0.07	0.04	0.1	0.0005	5
Swarthmore42	GPT-3.5	0.02	0.8	0.4	0.5	0.02	0.9	0.07	0.0008	3
UChicago30	GPT-3.5	0.02	0.2	0.1	1	0.02	0.1	0.03	0.003	1
Caltech36	LLAMA-3	0.06	0.1	0.08	1	0.07	0.04	0.02	1	5
Swarthmore42	LLAMA-3	0.02	0.9	0.09	1	0.02	0.9	0.02	1	3
UChicago30	LLAMA-3	0.02	0.2	0.02	1	0.02	0.07	0.007	1	1
Caltech36	Claude 3.5	0.05	0.3	0.1	1	0.05	0.2	0.03	0.9	5
Swarthmore42	Claude 3.5	0.02	0.9	0.3	0.9	0.01	1	0.02	0.9	3
UChicago30	Claude 3.5	0.02	0.2	0.2	0.3	0.02	0.2	0.01	0.9	1
Andorra	GPT-4 Mini	0.002	1	0	1	0.1	1	0.001	1	0.2
MobileD	GPT-4 Mini	0.03	0.3	0	1	0.08	3e-06	0.06	0.002	3
Andorra	GPT-3.5	0.002	1	0	1	0.1	1	0.002	1	0.2
MobileD	GPT-3.5	0.04	0.06	0	1	0.05	0.02	0.2	6e-29	4
Andorra	LLAMA-3	0.002	1	0	1	0.1	1	0.0009	1	0.2
MobileD	LLAMA-3	0.04	0.08	0	1	0.09	8e-07	0.04	0.06	4
Andorra	Claude 3.5	0.002	1	0	1	0.1	1	0.001	1	0.2
MobileD	Claude 3.5	0.04	0.04	0	1	0.2	5e-39	0.1	9e-19	4
					Recommendation System					
Caltech36	GPT-4 Mini	0.05	0.2	0.1	1	0.06	0.2	0.05	0.2	5
Swarthmore42	GPT-4 Mini	0.02	0.9	0.1	1	0.02	0.9	0.04	0.1	2
UChicago30	GPT-4 Mini	0.01	0.6	0.3	0.03	0.01	0.7	0.02	0.4	0.7
Caltech36	GPT-3.5	0.05	0.2	0.5	0.6	0.06	0.1	0.07	0.03	5
Swarthmore42	GPT-3.5	0.02	0.8	0.3	0.9	0.02	1	0.07	0.0005	3
UChicago30	GPT-3.5	0.01	0.5	0.5	0.002	0.01	0.6	0.02	0.08	0.9
Caltech36	LLAMA-3	0.06	0.2	0.1	1	0.07	0.06	0.03	0.9	5
Swarthmore42	LLAMA-3	0.01	1	0.2	1	0.01	1	0.02	1	1
UChicago30	LLAMA-3	0.006	1	0.2	0.4	0.007	1	0.005	1	0.3
Caltech36	Claude 3.5	0.05	0.2	0.2	1	0.04	0.4	0.08	0.02	5
Swarthmore42	Claude 3.5	0.01	1	0.2	1	0.008	1	0.05	0.02	2
UChicago30	Claude 3.5	0.006	1	0.4	0.02	0.006	1	0.01	0.6	0.5
Andorra	GPT-4 Mini	0.002	1	0	1	0.1	1	0.003	1	0.1
MobileD	GPT-4 Mini	0.03	0.6	0	1	0.07	0.0003	0.08	2e-05	2
Andorra	GPT-3.5	0.002	1	0	1	0.1	1	0.004	0.9	0.2
MobileD	GPT-3.5	0.05	0.01	0	1	0.04	0.08	0.07	0.0004	4
Andorra	LLAMA-3	0.002	1	0	1	0.1	1	0.003	1	0.2
MobileD	LLAMA-3	0.04	0.05	0	1	0.07	6e-05	0.08	9e-06	4
Andorra	Claude 3.5	0.002	1	0	1	0.1	1	0.005	0.7	0.2
MobileD	Claude 3.5	0.04	0.06	0	1	0.1	2e-17	0.1	6e-14	4

Table 3: Change in Graph Statistics for the experiments of Table 1. We report the KS statistic and the P-values for the following quantities (see [3] for more information on the statistics): (i) degree distribution, (ii) distribution of the sizes of strongly connected components, (iii) adjacency matrix spectrum, (iv) local clustering coefficient. The last column reports the percentage of new edges added. Adding $\leq 5\%$ of edges based on LLM decisions leaves global graph properties largely unchanged, with only occasional local clustering increases—more frequent under the Uniform strategy than the Recommendation System.

B.3.3 Average Marginal Effects

In Table 4 we report the average marginal effects (AMEs) per feature for the experiments of Table 1. Our analysis reveals that LLM-driven edge formation is consistently shaped by preferential attachment and homophily, with homophily often exhibiting the largest marginal effects – frequently exceeding 1.0 and reaching above 2.5 under the Recommendation System strategy. Preferential attachment is positive across all datasets and models, indicating a systematic tendency to link to high-degree nodes. Triadic closure effects are more variable, sometimes reinforcing local clustering and sometimes favoring cross-community connections, particularly under the Recommendation System. Compared to Uniform edge additions, the Recommendation System generally amplifies both preferential attachment and homophily, suggesting that recommendation-driven link formation intensifies these social-network-like biases.

		$\hat{\theta}_{PA}$		$\hat{\theta}_{H}$		$\hat{\theta}_{TC}$	
	Algorithm	Uniform	RecSys	Uniform	RecSys	Uniform	RecSys
Name	Model						
Caltech36	Claude 3.5	0.39*** (0.00)	0.57*** (0.00)	0.47*** (0.01)	1.62*** (0.01)	0.47*** (0.01)	0.18*** (0.01)
	GPT-3.5	0.18*** (0.00)	0.14*** (0.00)	0.61*** (0.00)	0.07*** (0.01)	-0.05*** (0.01)	-0.56*** (0.01)
	GPT-4 Mini	0.21*** (0.00)	0.17*** (0.00)	1.30*** (0.02)	1.85*** (0.00)	0.27*** (0.01)	0.27*** (0.00)
	LLAMA-3	0.11*** (0.00)	0.10*** (0.00)	0.96*** (0.01)	1.82*** (0.01)	0.33*** (0.00)	0.30*** (0.01)
Swarthmore42	Claude 3.5	0.29*** (0.00)	1.07*** (0.00)	0.60*** (0.01)	1.03*** (0.01)	0.44*** (0.00)	0.80*** (0.01)
	GPT-3.5	0.18*** (0.01)	0.13*** (0.00)	0.44*** (0.01)	0.10*** (0.00)	0.00 (0.01)	-0.08*** (0.00)
	GPT-4 Mini	0.12*** (0.00)	0.23*** (0.01)	1.01*** (0.01)	2.03*** (0.01)	0.26*** (0.00)	0.31*** (0.00)
	LLAMA-3	0.17*** (0.00)	0.05*** (0.00)	0.99*** (0.00)	1.52*** (0.01)	0.26*** (0.00)	0.69*** (0.01)
Uchicago30	Claude 3.5	0.35*** (0.00)	0.66*** (0.00)	0.64*** (0.00)	1.47*** (0.00)	0.32*** (0.00)	0.15*** (0.00)
	GPT-3.5	0.21*** (0.00)	0.13*** (0.00)	0.45*** (0.00)	-0.08*** (0.01)	-0.02*** (0.00)	0.17*** (0.01)
	GPT-4 Mini	0.13*** (0.00)	0.27*** (0.00)	1.03*** (0.01)	2.82*** (0.01)	0.24*** (0.00)	-0.60*** (0.00)
	LLAMA-3	0.21*** (0.00)	0.19*** (0.00)	1.27*** (0.01)	1.97*** (0.01)	0.16*** (0.00)	0.37*** (0.01)
MobileD	Claude 3.5	0.43*** (0.01)	1.39*** (0.01)	-1.47*** (0.01)	-0.66*** (0.00)	0.68*** (0.00)	0.12*** (0.01)
	GPT-3.5	0.83*** (0.00)	1.30*** (0.00)	-0.74*** (0.01)	-0.27*** (0.01)	-0.02*** (0.00)	-0.70*** (0.01)
	GPT-4 Mini	0.89*** (0.01)	2.06*** (0.01)	-0.55*** (0.01)	-0.32*** (0.01)	0.56*** (0.01)	0.44*** (0.01)
	LLAMA-3	0.66*** (0.00)	1.02*** (0.01)	0.23*** (0.01)	0.75*** (0.01)	0.45*** (0.00)	0.26*** (0.00)
Andorra	Claude 3.5	0.40*** (0.00)	0.30*** (0.00)	1.45*** (0.01)	1.40*** (0.01)	-0.11*** (0.00)	-0.32*** (0.00)
	GPT-3.5	0.43*** (0.00)	0.25*** (0.02)	0.17*** (0.01)	-0.06*** (0.01)	-0.19*** (0.00)	-0.35*** (0.01)
	GPT-4 Mini	0.31*** (0.00)	0.08*** (0.00)	2.00*** (0.03)	2.54*** (0.01)	-0.05*** (0.01)	-0.45*** (0.00)
	LLAMA-3	0.19*** (0.00)	0.33*** (0.00)	1.92*** (0.01)	2.24*** (0.01)	-0.02* (0.01)	-0.08*** (0.00)

Note: *: $P < 0.05$, **: $P < 0.01$, ***: $P < 0.001$

Table 4: AMEs for the discrete choice models of Table 1.

B.4 Robustness of Results to Temperature

B.4.1 Regression Coefficients

In Table 5, we report the regression coefficient for the regression in the real-world network data for all temperatures and GPT-4 (gpt-4-1106-preview). The first column corresponds to the temperature, the next three columns correspond to the fitted coefficients from the regression model of Section 1.C (also shown in Figure 5) accompanied by the standard errors (in parentheses) and the P-values indicated by stars (the null hypothesis corresponds to the parameters being set to 0). Next, LL corresponds to the log-likelihood of the fitted model, and AIC corresponds to the Akaike Information Criterion. Finally, we report the percent change in the accuracy compared to random guessing, the percent change in the average path length (as a measure of the small-world phenomenon), and the clustering coefficient (as a measure of the small-world phenomenon and the triadic closure), as well as the t-statistic for the change in modularity (Q) between the ground truth network dataset (before the edge deletions) and the network after the network formation process.

We observe that $\hat{\theta}_{\text{H}} > \hat{\theta}_{\text{TC}} > \hat{\theta}_{\text{PA}} > 0$ accross all settings. LLM agents do better than random guessing, reinforce the small-world phenomenon, and weaken the triadic closure, though the changes are very small, 0-1% change for the average path length and up to 10% change for the clustering coefficient. Finally, the community structure is strengthened after new links are formed.

Temp.	$\hat{\theta}_{\text{PA}}$	$\hat{\theta}_{\text{H}}$	$\hat{\theta}_{\text{TC}}$	LL	AIC	% Change Acc.	% Change L	% Change C	ΔQ (t-stat)
Caltech36 (769 nodes, 33,312 edges)									
0.5	0.41*** (0.01)	1.95*** (0.02)	0.59*** (0.01)	-1,377.47	2,762.94	171.8	-0.008	-9.94	3.45**
1.0	0.36*** (0.005)	1.85*** (0.02)	0.58*** (0.01)	-1,435.07	2,878.13	179.6	-0.18	-11.08	3.49**
1.5	0.36*** (0.006)	1.72*** (0.01)	0.55*** (0.007)	-1,522.47	3,052.94	127.6	-0.06	-11.46	3.37**
Swarthmore42 (1,659 nodes, 12,2100 edges)									
0.5	0.18*** (0.003)	1.62*** (0.006)	0.65*** (0.002)	-2,838.33	5,684.66	124.2	0.01	-11.46	7.42***
11.0	0.26*** (0.002)	1.70*** (0.008)	0.58*** (0.003)	-2,927.99	5,863.97	91.6	-0.10	-4.25	1.96*
1.5	0.19*** (0.004)	1.50*** (0.008)	0.59*** (0.002)	-3,139.42	6,286.83	87.39	-0.20	-4.52	4.03***
UChicago30 (6,591 nodes, 416,206 edges)									
0.5	0.23*** (0.001)	2.00*** (0.005)	0.41*** (0.002)	-3,444.33	6,896.67	217.2	-0.24	-2.52	7.46*** [0.34]
1.0	0.23*** (0.002)	1.98*** (0.004)	0.38*** (0.001)	-3,578.18	7,164.36	219.2	-0.12	-2.66	9.56*** [1.05]
1.5	0.22*** (0.004)	1.78*** (0.008)	0.41*** (0.002)	-2,033.49	4,074.98	222.4	-0.17	-2.42	10.19*** [0.24]

Notes $\hat{\theta}_{\text{PA}}$ = Coefficient of log degree, $\hat{\theta}_{\text{H}}$ = Coefficient of log # of common attributes, $\hat{\theta}_{\text{TC}}$ = Coefficient of log # common neighbors
LL = Log-likelihood, AIC = Akaike Information Criterion
Acc. = Accuracy, L = Average Path Length, C = Average Clustering Coefficient, ΔQ (t-stat) = Modularity change t-statistic
$^{*}: P < 0.05$, $^{**}: P < 0.01$, $^{***}: P < 0.001$

Table 5: Multinomial logit coefficients for three networks from the Facebook100 dataset and GPT-4 (gpt-4-1106-preview). The standard errors of the estimates are shown in parentheses. The null hypothesis corresponds to the respective parameter being equal to 0. We report the percent change in accuracy, average path length, and average clustering coefficient compared to the initial network (before the deletion of edges). For the change in modularity, we run the Louvain algorithm ten times and perform a t-test with the resulting modularities. For the UChicago30 dataset, we report the t-statistic value in the subgraph induced by the 2,000 sampled nodes, since the newly added edges would have a very small effect on the change in the community structure if we were to measure it in the whole network. We also report the modularity change (t-statistic) of the whole graph inside brackets.

B.4.2 Change in Graph Statistics due to Different Temperatures

Further, to measure the changes in the network statistics, we use the metrics presented in [3] to quantify the changes. Specifically, we measure the Kolmogorov-Smirnov statistic and the corresponding P-value for the degree distribution, the distribution of the sizes of the connected components, the distribution of the singular values of the adjacency matrix, and the distribution of the local clustering coefficient, (CC) for the real-world Facebook100 networks and the gpt-4-1106-preview model (the results are similar for the other networks and models we examined). Except for the changes in the distribution of the sizes of the connected components for UChicago30, we find that most KS statistics are negligible and the corresponding P-values are large (e.g., $P \gg 0.5$), indicating that most network statistics are not affected. Table 6 summarizes the results:

Name	Temp	Degrees (KS)	(P-value)	Sizes of CCs (KS)	(P-value)	Spectrum (KS)	(P-value)	Local CC (KS)	(P-value)
Caltech36	0.5	0.0481	0.336	0.125	0.999	0.0546	0.202	0.0234	0.984
	1.0	0.0481	0.336	0.176	0.926	0.0559	0.181	0.0325	0.811
	1.5	0.0481	0.336	0.111	1	0.0559	0.181	0.0351	0.731
Swarthmore42	0.5	0.0229	0.777	0.2	0.987	0.0151	0.992	0.0127	0.999
	1.0	0.0217	0.83	0.2	0.987	0.0133	0.999	0.0133	0.999
	1.5	0.0211	0.854	0.2	0.987	0.0157	0.987	0.0175	0.962
UChicago30	0.5	0.00228	1	0.81	3.4e-08 (***)	0.00303	1	0.0188	0.194
	1.0	0.00228	1	0.805	4.62e-08 (***)	0.00288	1	0.0184	0.217
	1.5	0.00789 (***)	0.986	0.873	3.86e-11 (***)	0.00819	0.98	0.0188	0.194

Table 6: Change in graph statistics for the GPT-4 model (gpt-4-1106-preview) and the Facebook100 data by the metrics outlined in [3]. The results for the other datasets, models, and temperatures are similar. (***) denotes $P < 0.001$.

B.5 Robustness of Results to Large Context Windows

We perform experiments with the large-context model gpt-4.1-mini. We set the temperature to 0.5. Table 7 shows the effect sizes. We observe that for social networks (Caltech36, Swarthmore42, UChicago30) homophily still remains the dominant force for large context windows and that in most cases $\hat{\theta}_H > \hat{\theta}_{TC} > \hat{\theta}_{PA} > 0$, agreeing with the results of Table 1. Additionally, for the MobileD network, we observe heterophily ($\hat{\theta}_H < 0$). On the other hand, we observe that for the Andorra dataset, for larger contexts, the triadic closure has a positive effect ($\hat{\theta}_{TC} > 0$; $P < 0.001$) compared to a negative weight in Table 1 ($P < 0.001$).

Dataset	Preferential Attachment ($\hat{\theta}_{\mathrm{PA}}$)	Homophily ($\hat{\theta}_{\mathrm{H}}$)	Triadic Closure ($\hat{\theta}_{\mathrm{TC}}$)	Log Likelihood	AIC
		$A = 50$			
Caltech36	0.30*** (0.002)	2.74*** (0.01)	0.25*** (0.006)	-2,080.91	4,169.83
Swarthmore42	0.17*** (0.002)	2.23*** (0.004)	0.40*** (0.002)	-2,708.70	5,425.41
UChicago30	0.16*** (0.004)	2.04*** (0.005)	0.49*** (0.002)	-2,553.28	5,114.57
Andorra	0.27*** (0.01)	6.32*** (0.02)	0.30*** (0.01)	-1,762.45	3,532.89
MobileD	0.27** (0.005)	-0.84*** (0.02)	1.53*** (0.002)	-2,359.88	4,727.77
		$A = 100$			
Caltech36	0.35*** (0.003)	3.23*** (0.02)	0.26*** (0.002)	-2,450.46	4,908.91
Swarthmore42	0.15** (0.005)	2.51*** (0.004)	0.33*** (0.002)	-3,522.14	7,052.27
UChicago30	0.16*** (0.003)	2.66*** (0.004)	0.40*** (0.003)	-3,100.58	6,209.16
Andorra	0.23*** (0.006)	6.72*** (0.04)	0.56*** (0.002)	-1,838.66	3,685.33
MobileD	0.32*** (0.004)	-0.23 (0.008)	1.58*** (0.004)	-2,848.65	5,705.30

Note: *: $P < 0.05$, **: $P < 0.01$, ***: $P < 0.001$

Table 7: Robustness of results to large contexts. Experiments have been performed with gpt-4.1-mini with context windows $A \in \{50, 100\}$. The temperature has been set to 0.5.

C Network Evolution and Omitted Simulations

Here we depict the evolution of the networks generated by the LLM agents, as well as omitted simulations.

C.1 Principle 1: Preferential Attachment

Network Evolution

We plot the evolution of the LLM-based preferential attachment networks at three timesteps, together with the degree distribution alongside the degree distribution of a BA graph with the same number of nodes. We observe that for the temperature being 0.5, we have a core-periphery-like formation which diverges from the BA model, whereas for the temperature being 1.5, the network has the same degree distribution as the BA model.

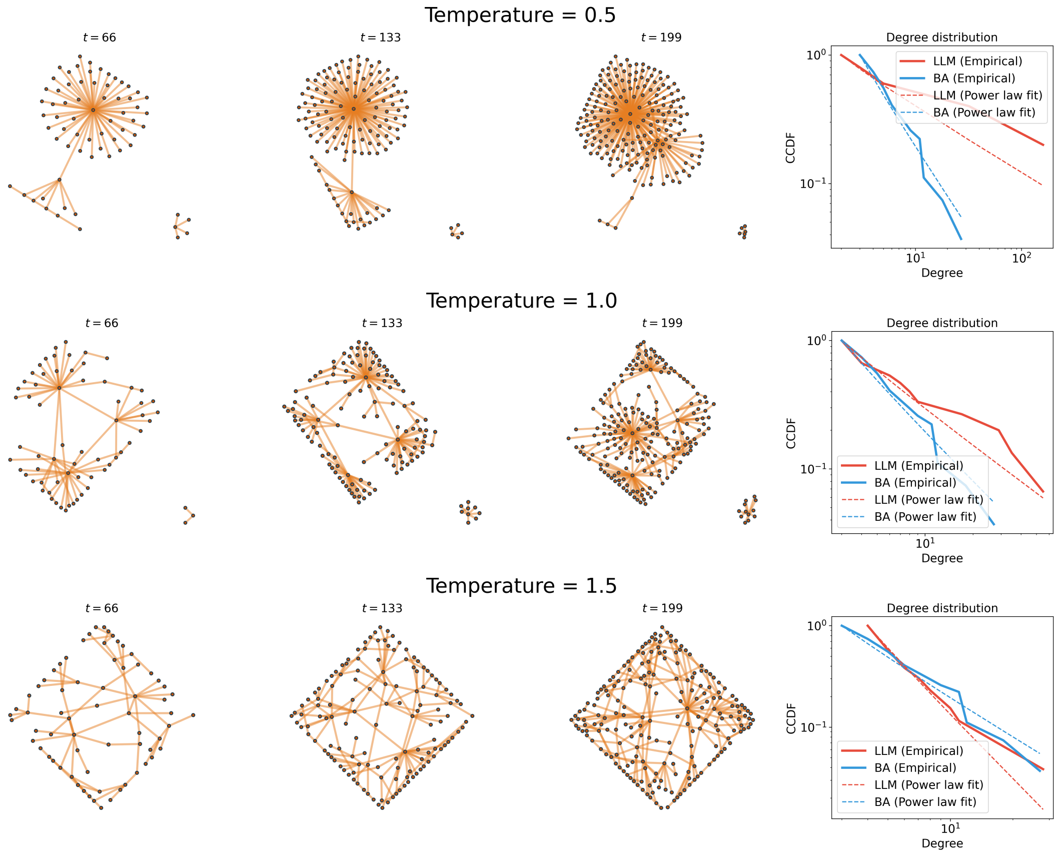

Figure 3: Dynamic evolution of networks created based on Principle 1.

Simulations with Degree Information

In Figure 4 we provide the results with degree-information only. We observe that the agents form connections around high-degree nodes only (see Figure 4). The same result (star-like networks) holds for the other LLM models and temperatures.

C.2 Principle 2: Triadic Closure

Network Evolution

We plot the evolution of the LLM-generated networks based on the triadic closure principle, together with the transitivity measure and the algebraic connectivity (which corresponds to the second-smallest

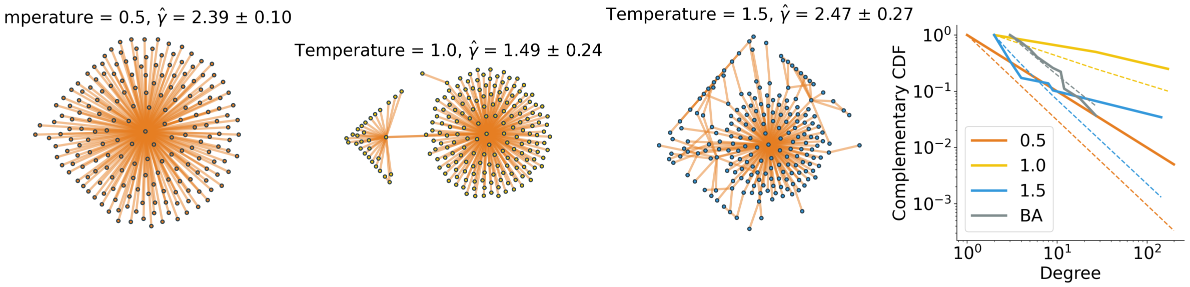

Figure 4: Results for Principle 1 (preferential attachment): We display simulated networks comprising 200 nodes across different temperatures. For the degree-based simulations, node degree data $\{d_{j,t} : j \in V_t\}$ was provided (V_t corresponds to the vertex set of the network G_t at round t). With degree information only, the networks form more unrealistic star-like structures, diverging from scale-free configurations and more closely mirroring a core-periphery network structure.

eigenvalue of the graph Laplacian). We observe that the algebraic connectivity gradually increases as new edges between the clusters are created. Specifically, the algebraic connectivity reaches a higher value for higher temperatures, indicating the more frequent creation of new intra-cluster edges. Moreover, we observe that the transitivity initially increases and then decreases until it reaches its final value.

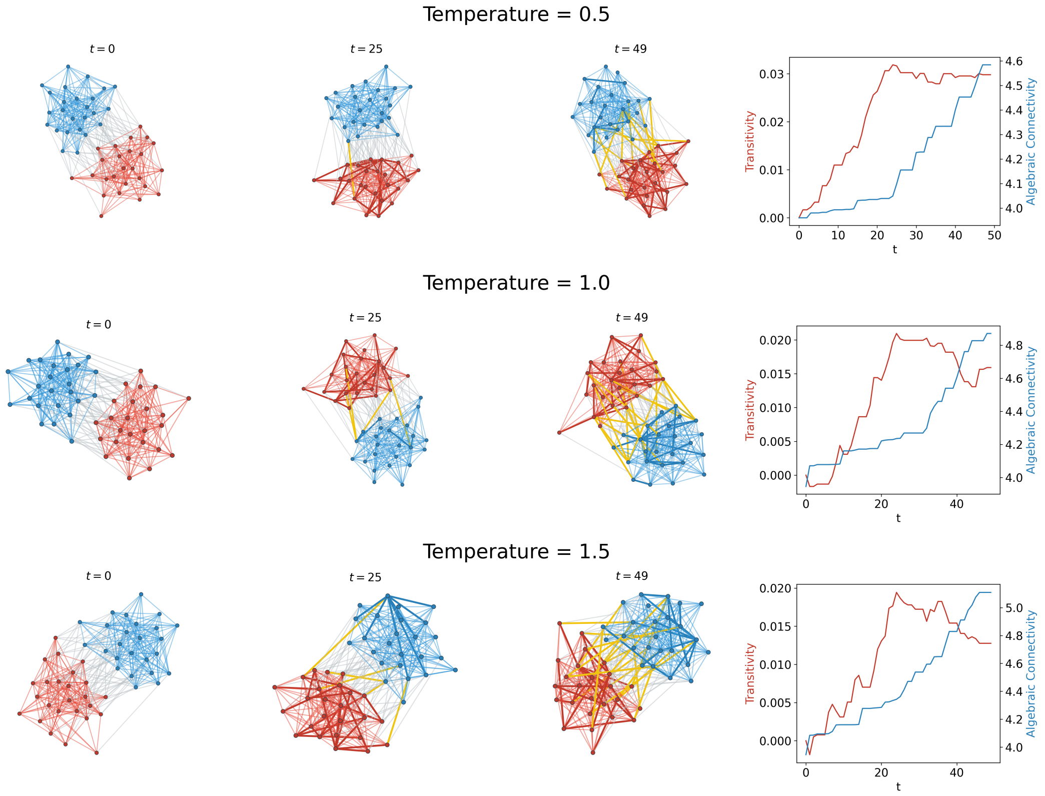

Figure 5: Dynamic evolution of networks created based on Principle 2.

Simulations with the Number of Common Neighbors

Instead of giving the neighborhood information, the simulations presented in Figure 2 use the number of common neighbors. We observe behavior similar to Figure 2.

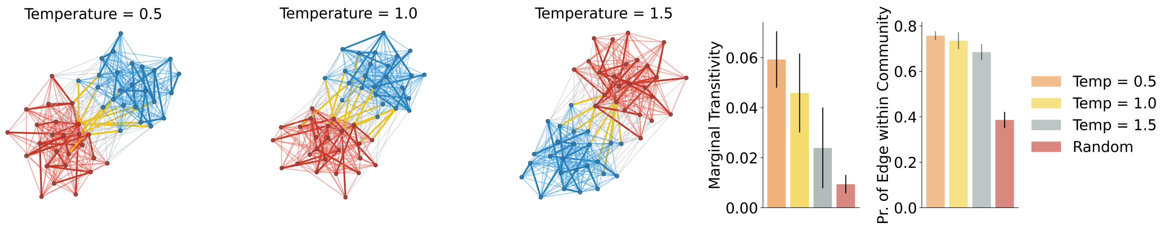

Figure 6: Results for Principle 2 (triadic closure). The figure shows the same networks as in Principle 2 with the only change that instead of the intersection of neighborhoods between the query node and each alternative, we provide the number of common neighbors (i.e., the size of the intersection) between the query node and each alternative. Similarly, we observe that the probability of forming an edge within the same community and the marginal transitivity, which indicates triadic closure, is significantly larger than randomly creating links ($P < 0.001$, t-test). The error bars correspond to 95% confidence intervals.

C.3 Principle 5: Small-World Phenomenon

Figure 7 shows LLM-generated small-world networks for $\beta \in \{0.25, 0.5, 0.75\}$ and compares them with the Watts-Strogatz networks with the same parameters.

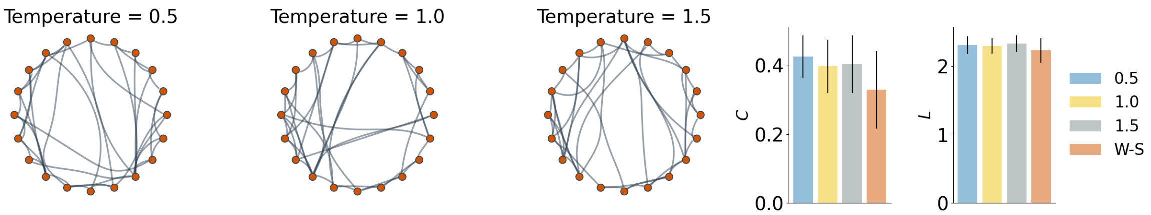

(a) $\beta = 0.25$

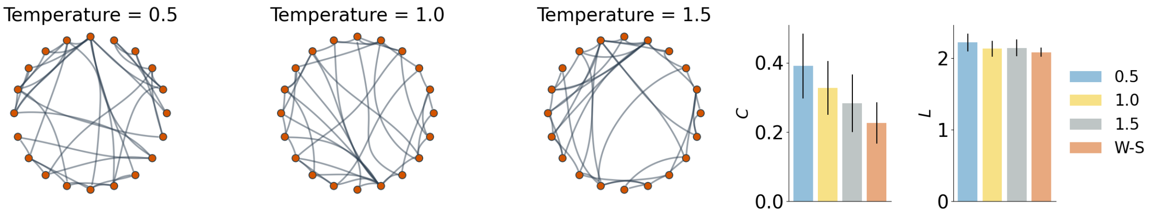

(b) $\beta = 0.5$

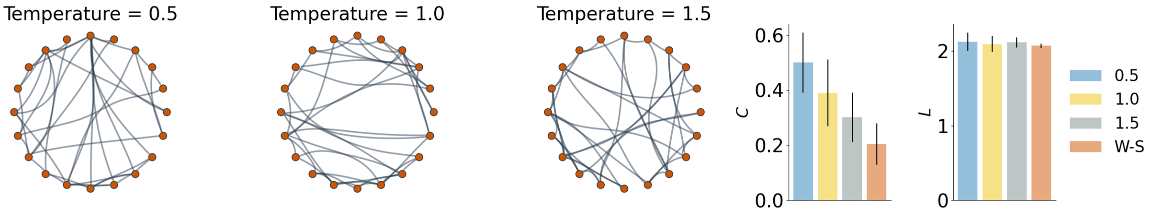

(c) $\beta = 0.75$

Figure 7: Simulation results for Principle 5 (small world). Network instances for the networks created according to Principle 5 using the altered Watts-Strogatz Model for node count $n = 50$, average degree $k = 5$, rewriting probability $\beta \in \{0.25, 0.5, 0.75\}$, together with plots of the **average clustering coefficient** C and the **average shortest path length** L. The comparison is made with respect to a Watts-Strogatz graph with $n = 50, k = 5, \beta \in \{0.25, 0.5, 0.75\}$. The error bars correspond to 95% confidence intervals. The results are compared against the Watts-Strogatz model with the same parameters k and β as a null model. The t-test comparing L and C for the LLM-generated networks and Watts-Strogatz networks yields $P > 0.05$ (Bonferroni correction for two tests).

D Chain-of-Thought Experiments

We experiment with Chain-of-Thought (CoT) reasoning [6]. To induce CoT reasoning, we ask the LLM agents to output the reason and then their choice (i.e., by reversing the order of `reason` and `name` in Algorithm 2). The resulting prompt can be found at Algorithm 4. In the following figures, we show the results from the same experiments as the ones we presented in the main text with the difference that CoT is used.

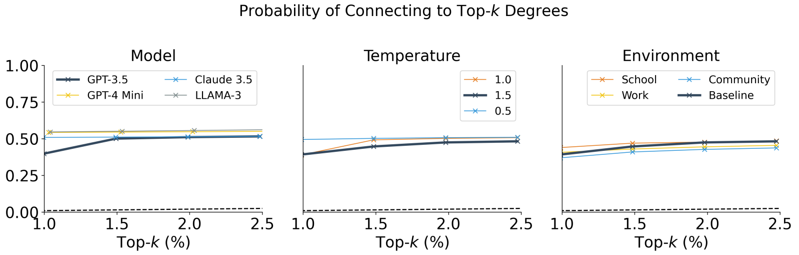

(a) Probability of connecting to top-k nodes for different models, temperatures, and environments

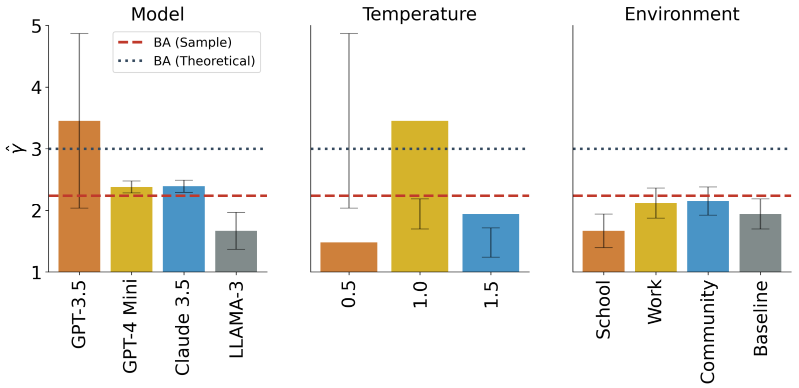

(b) Power law fits ($\hat{\gamma}$) and standard errors for different models, temperatures, and environments

Figure 8: Results for Principle 1 with CoT reasoning (preferential attachment) The multi-LLM setup was given neighborhood information $\{N_{j,t} : j \in V_t\}$. **Top:** Probability of connecting to top-k-degree nodes for varying model (temperature is fixed to 1.0 and environment to baseline), temperature (model fixed to GPT-3.5 and environment to baseline), and environment (model fixed to GPT-3.5 and environment temperature to 1.5) for networks generated according to Principle 1 with $n = 200$ nodes. **Bottom:** Power Law exponents and standard errors for varying model, temperature, and environment.

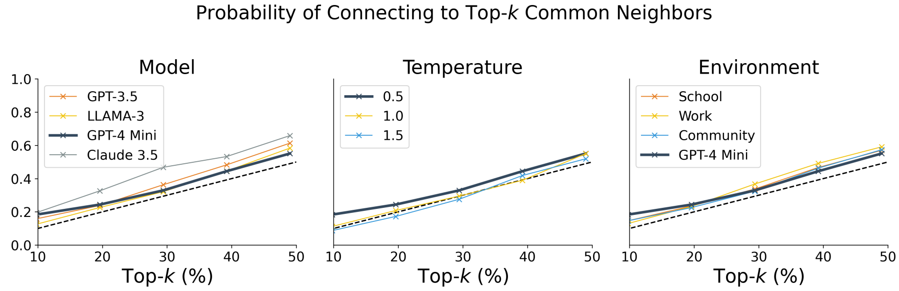

(a) Probability of connecting to top-k for different models, temperatures, and environments

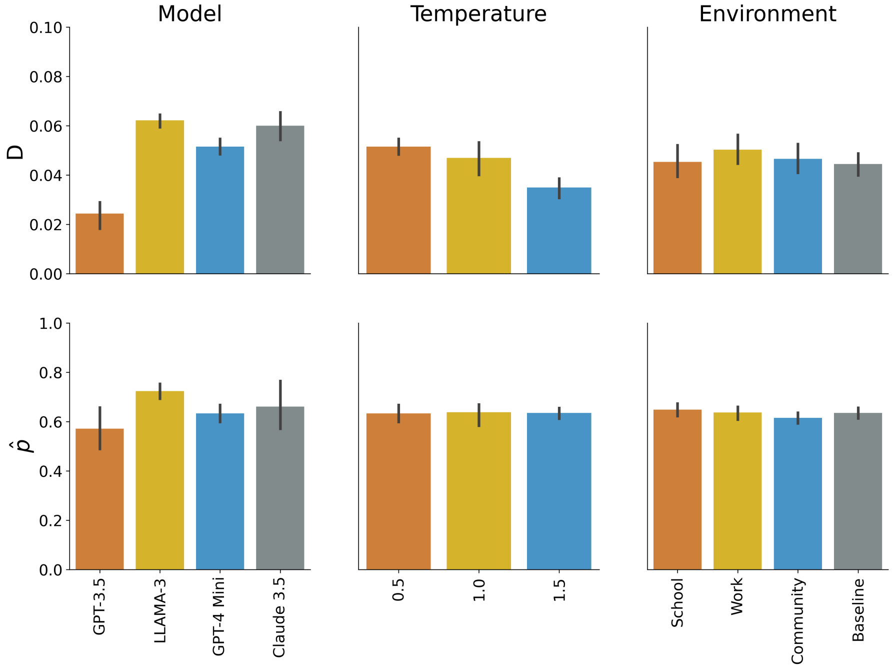

(b) Marginal transitivity (D) and probability of an edge within a community ($\hat{p}$) for different models, temperatures, and environments

Figure 9: Results for Principle 2 with CoT reasoning (triadic closure). Top: Probability of connecting to top-k nodes (in terms of common neighbors) for varying model (temperature is fixed to 1.0 and environment to baseline), temperature (model fixed to GPT-4 Mini and environment to baseline) and environment (model fixed to GPT-4 Mini and environment temperature to 0.5) for networks generated according to Principle 2 (n = 50, 10 simulations for each model, environment and temperature). **Bottom:** Marginal transitivity (D) and probability of an edge within a community ($\hat{p}$) for networks generated according to Principle 2 in different models, temperatures, and environments.

Algorithm 4 Example prompt regarding social network data with Chain-of-Thought reasoning. Note that compared to Algorithm 1 the order of the fields `name` and `reason` in the output format is reversed.

```
# Task
You are located in a school. Your task is to select a set of people to be friends
 with.

# Profile
Your profile is given below after chevrons:
<PROFILE>
    {
        "name" : "Person 0",
        "favorite subject" : "Chemistry",
        "neighbors" : ["Person 3", "Person 432", "Person 4", "Person 3", "Person
         32"]
    }
</PROFILE>

# Candidate Profiles
The candidate profiles to be friends with are given below after chevrons:

<PROFILES>
[
    {
        "name" : "Person 1",
        "favorite subject" : "Mathematics",
        "neighbors" : ["Person 3", "Person 4", "Person 23", "Person 65"]
    },
    {
        "name" : "Person 33",
        "favorite subject" : "History",
        "neighbors" : ["Person 342", "Person 2", "Person 12"]
    }, ...
]

</PROFILES>

# Output
The output should be given a list of JSON objects with the following structure

[
    {{
        "reason" : reason for selecting the person,
        "name" : name of the person you selected
    }}, ...
]

# Notes
- The output must be a list of JSON objects ranked in the order of preference.
- You can make at most 1 selection.
```

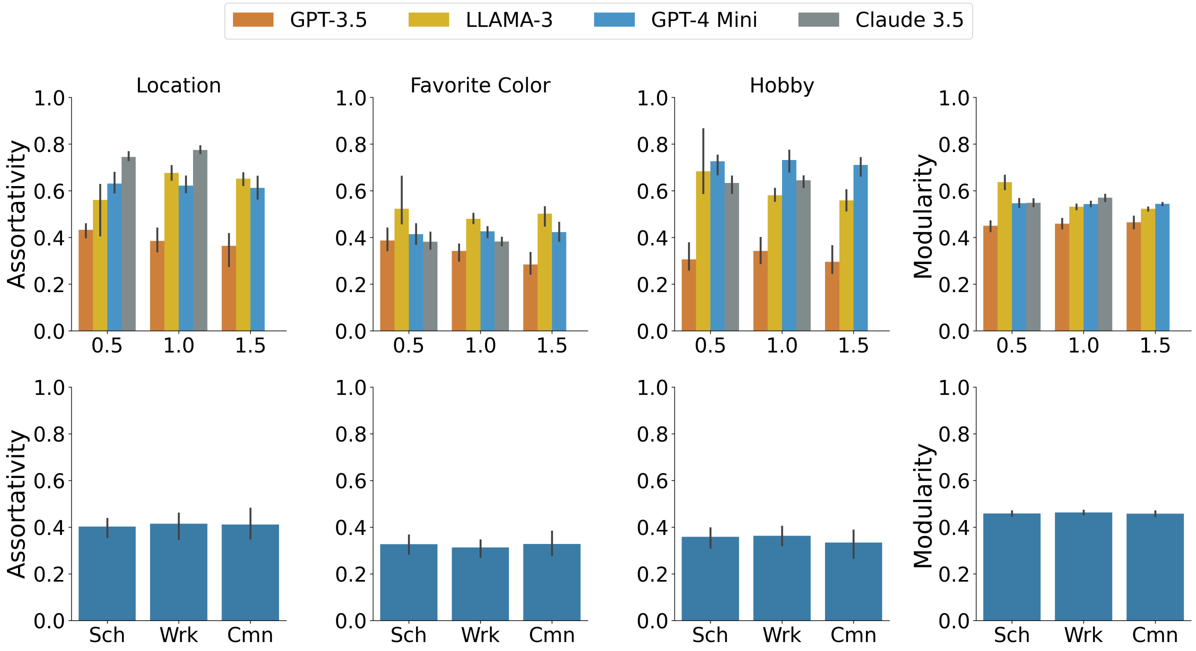

(a) Assortativity and Louvain Modularity with different LLM models and environments

Figure 10: Results for Principle 3 (Homophily) and Principle 4 (Community structure due to homophily) with CoT reasoning. Top: Assortativities and Louvain modularity according to Principle 3 ($n = 50$, 5 simulations for each row) in different environments (school, work, community) using different models. The statistical significance is $P < 0.0003$ for all t-tests (comparing with 0, Bonferroni correction for three tests).

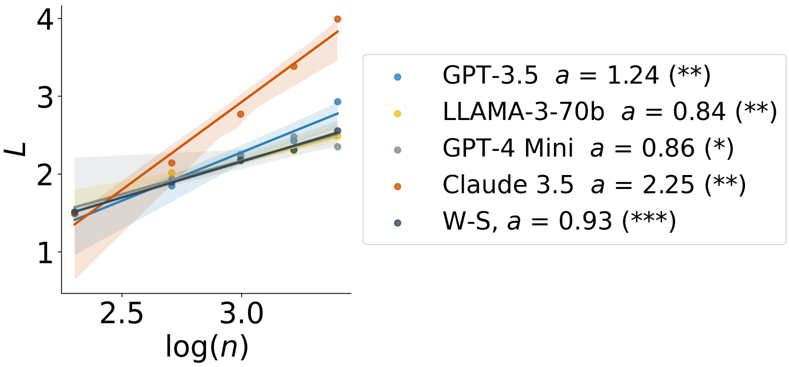

(a) Regression plot for different models and environments for $\beta = 0.25$ and $k = 5$.

Figure 11: Fitted results for Principle 5 with CoT reasoning (small world). Regression plot for the relation $L \sim \log(n)$ for different LLM models for $\beta = 0.25$ and $k = 5$. The legend shows the effect size (a) and the P-value. (*: $P < 0.0025$; **: $P < 0.005$, and ***: $P < 0.0005$, Bonferroni correction for two tests).

References

[1] Shikha Bordia and Samuel Bowman. Identifying and Reducing Gender Bias in Word-Level Language Models. In *Proceedings of the 2019 Conference of the North American Chapter of the Association for Computational Linguistics: Student Research Workshop*, pages 7–15, 2019.

[2] Hadas Kotek, Rikker Dockum, and David Sun. Gender Bias and Stereotypes in Large Language Models. In *Proceedings of The ACM Collective Intelligence Conference*, pages 12–24, 2023.

[3] Jure Leskovec and Christos Faloutsos. Sampling from Large Graphs. In *Proceedings of the 12th ACM SIGKDD International Conference on Knowledge Discovery and Data Mining*, pages 631–636, 2006.

[4] Amanda L Traud, Peter J Mucha, and Mason A Porter. Social Structure of Facebook Networks. *Physica A: Statistical Mechanics and its Applications*, 391(16):4165–4180, 2012.

[5] Jesse Vig, Sebastian Gehrmann, Yonatan Belinkov, Sharon Qian, Daniel Nevo, Yaron Singer, and Stuart Shieber. Investigating Gender Bias in Language Models using Causal Mediation Analysis. *Advances in Neural Information Processing Systems*, 33:12388–12401, 2020.

[6] Jason Wei, Xuezhi Wang, Dale Schuurmans, Maarten Bosma, Ed Chi, Quoc Le, and Denny Zhou. Chain of thought prompting elicits reasoning in large language models. *arXiv preprint arXiv:2201.11903*, 2022.

[7] Yuan Yuan, Ahmad Alabdulkareem, and Alex Pentland. An Interpretable Approach for Social Network Formation among Heterogeneous Agents. *Nature Communications*, 9(1):4704, 2018.
